# Minimal Functionalization of Ruthenium Compounds with Enhanced Photoreactivity against Hard-to-Treat Cancer Cells and Resistant Bacteria

**DOI:** 10.1021/acs.inorgchem.4c02235

**Published:** 2024-07-23

**Authors:** Geângela de Fátima Sousa Oliveira, Florencio Sousa Gouveia, Alexandre Lopes Andrade, Mayron Alves de Vasconcelos, Edson Holanda Teixeira, Marcos V. Palmeira-Mello, Alzir A. Batista, Luiz Gonzaga de França Lopes, Idalina Maria Moreira de Carvalho, Eduardo Henrique Silva Sousa

**Affiliations:** † Laboratório de Bioinorgânica, Departmento de Química Orgânica e Inorgânica, Universidade Federal do Ceará, Fortaleza 60440-900, Brazil; ‡ Laboratório Integrado de Biomoléculas, Departamento de Patologia e Medicina Legal, Universidade Federal do Ceará, Fortaleza, Ceará 60441-750, Brazil; § Faculdade de Educação de Itapipoca, Universidade Estadual do Ceará, Itapipoca, Ceará 62500-000, Brazil; ∥ Departamento de Química, Universidade Federal de São Carlos, PO Box 676, São Carlos, São Paulo 13565-905, Brazil

## Abstract

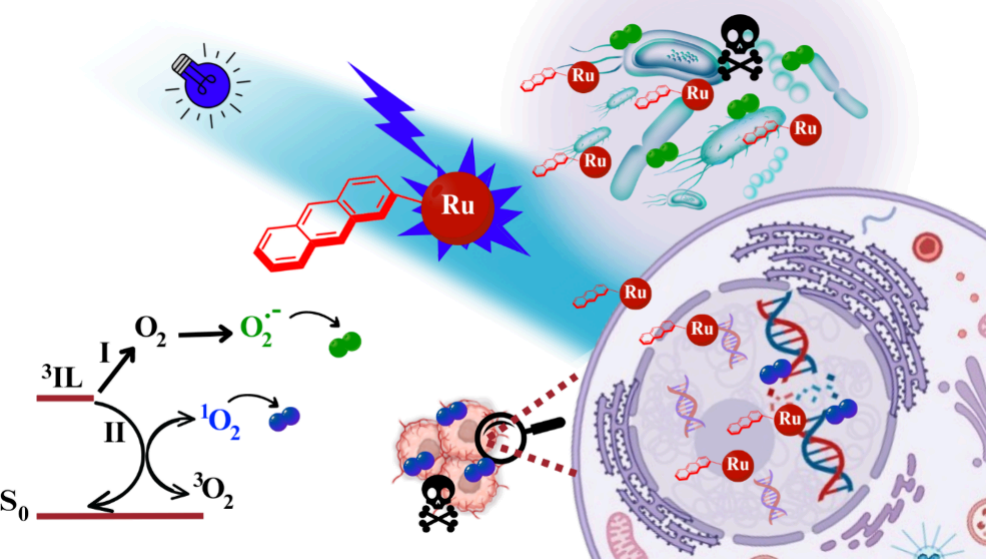

Metallocompounds have emerged as promising new anticancer agents, which can also exhibit properties to be used in photodynamic therapy. Here, we prepared two ruthenium-based compounds with a 2,2′-bipyridine ligand conjugated to an anthracenyl moiety. These compounds coded **GRBA** and **GRPA** contain 2,2′-bipyridine or 1,10-phenathroline as auxiliary ligands, respectively, which provide quite a distinct behavior. Notably, compound **GRPA** exhibited remarkably high photoproduction of singlet oxygen even in water (ϕ_Δ_ = 0.96), almost twice that of **GRBA** (ϕ_Δ_ = 0.52). On the other hand, this latter produced twice more superoxide and hydroxyl radical species than **GRPA**, which may be due to the modulation of their excited state. Interestingly, **GRPA** exhibited a modest binding to DNA (*K*_b_ = 4.51 × 10^4^), while **GRBA** did not show a measurable interaction only noticed by circular dichroism measurements. Studies with bacteria showed a great antimicrobial effect, including a synergistic effect in combination with commercial antibiotics. Besides that, **GRBA** showed very low or no cytotoxicity against four mammalian cells, including a hard-to-treat MDA-MB-231, triple-negative human breast cancer. Potent activities were measured for **GRBA** upon blue light irradiation, where IC_50_ of 43 and 13 nmol L^–1^ were seen against hard-to-treat triple-negative human breast cancer (MDA-MB-231) and ovarian cancer cells (A2780), respectively. These promising results are an interesting case of a simple modification with expressive enhancement of biological activity that deserves further biological studies.

## Abstract 2

Anthracene-based functionalization of polypyridine ruthenium compounds originated the distinct profile of photogeneration of ROS (^1^O_2_ and OH^**·**^/O_2_^**·**-^) and also performance on cancer versus bacterial cells. There is over 280-fold photoactivation along with nanomolar activity against hard-to-treat triple-negative human breast cancer (MDA-MB-231), which has opened great opportunities.

## Introduction

During the last decades, ruthenium complexes containing polypyridine ligands have been widely studied due to their many exciting and diverse properties (e.g., photophysical and photochemical processes, high stability, and reactivity).^1−3^ Some of these ruthenium complexes are composed of 2,2′-bipyridine (bpy) or 1,10-phenanthroline (phen) ligands. These ligands can originate stable metal complexes along with interesting chemical and biological properties. Despite the expressive number of combinations and modifications of ligands in ruthenium compounds reported in the literature, it seems there are still many remarkable properties to be unveiled.^4−6^ This indicates clearly how many opportunities are still available for exploration.

One of the areas of investigation in fast growth is in the development of ruthenium-based anticancer compounds.^7^ Currently, there are a series of these compounds with great antiproliferative effects against many cancer cells such as lung, ovarian, breast, and colon cancer.^8,9^ This activity has been attributed to multiple mechanisms of action of the metallocompounds, including the induction of apoptosis (programmed cell death) and inhibition of angiogenesis (formation of new blood vessels).^10−12^ In addition to their antitumor activity, several of these compounds have also unique photochemical and electrochemical properties that can be further explored. These properties are mostly due to a suitable combination of ruthenium and polypyridine ligands that allow light absorption over a wide range of wavelengths along with long-lived excited states and induced electron transfer processes as well.^13−15^

Although ruthenium complexes with polypyridine ligands have great potential in various applications, there are still many problems to be solved before these compounds can be used in clinical therapy and other applications.^16,17^ One of the challenges includes optimizing the potency and selectivity of these compounds, as well as understanding their mechanisms of biological activity. Ruthenium complexes containing anthracene-modified ligands have attracted interest in cancer research due to their rigid aromatic shape and photophysics properties.^11,18,19^ The combination of these properties has allowed their use in metal complexes as photosensitizers,^11,18,20^ chemosensors,^21^ DNA intercalators,^22^ and chemotherapeutic agent.^22,23^ These metal complexes containing such organic chromophores usually belong to a class of therapeutic agents known in photodynamic therapy (PDT).^24^

PDT is a therapeutic approach that uses photosensitizers to induce the selective death of tumor cells through photogeneration of reactive oxygen species (ROS). Ruthenium complexes have been shown to outperform many other types of photosensitizers, such as porphyrin derivatives, due to their high luminescence quantum efficiency and energy transfer properties. These properties may allow the metal complexes to induce selective cancer cell death with greater efficacy and lower toxicity compared to other photosensitizing agents.^3,25^

Currently, a polypyridine ruthenium complex, TLD-1433, is in clinical trial phase II with promising treatment of nonmuscle invasive bladder cancer using PDT.^3^ Energy transfer is a key property of ruthenium complexes containing polypyridine ligands and anchored chromophores. Anthracene is an energy-donating chromophore that can transfer energy to a bipyridine ligand of the ruthenium complex upon light excitation.^20^ This energy transfer process can generate reactive oxygen species (ROS), such as singlet oxygen, which can damage DNA and proteins inside a tumor cell. Preclinical studies have shown that ruthenium complexes containing polypyridine ligands and anthracene exhibit antitumor activity in various tumor cell lines, including breast, prostate, and lung cancer opening broad opportunities for the development of new treatments.^11,18^ Beyond cancer therapy, photodynamic antimicrobial chemotherapy (PACT) has emerged with many opportunities considering the major threat of resistance that is faced globally.^26^ This issue has opened new avenues for systems first thought for cancer, and a series of properties have been investigated, including singlet oxygen photogeneration and DNA binding/cleavage, along with antimicrobial and cytotoxicity studies using cancer cell lines.

**Figure 1:**
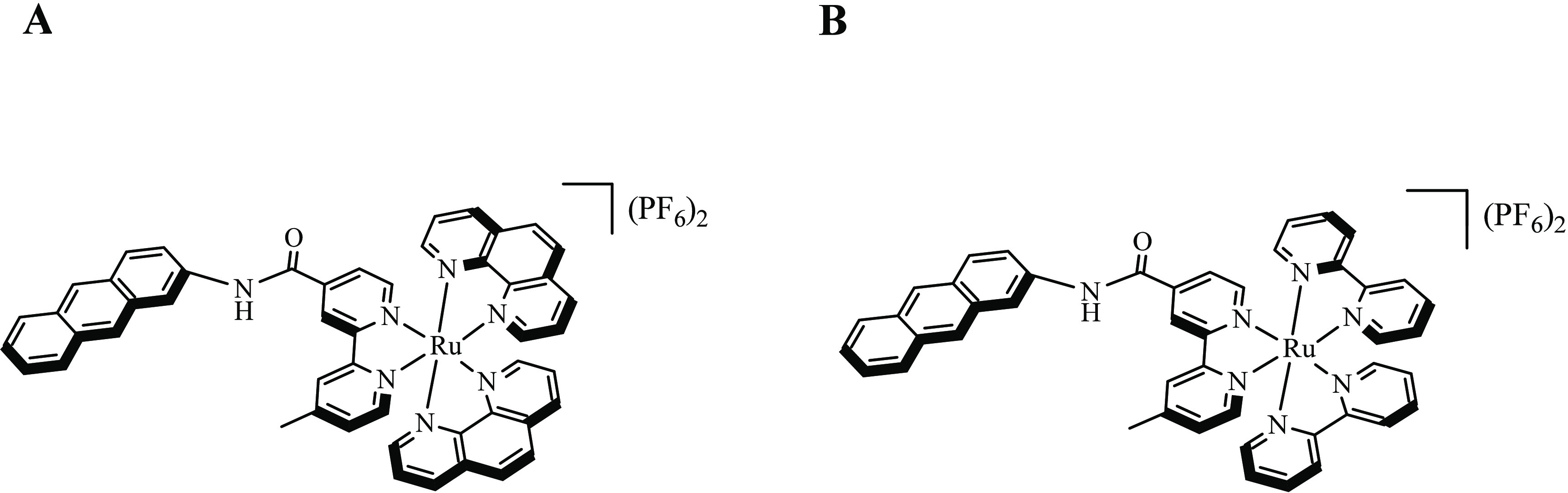
Structures of **GRPA** (A) and **GRBA** (B).

Previously, we reported some studies investigating ruthenium complexes containing a bipyridine ligand conjugated to an anthracenyl moiety. The energy gap between ^3^MLCT of the Ru(bpy) moiety and ^3^IL (triplet intraligand state) of the anthracenyl pendant ligand is in the order of 1700 cm^–1^, allowing an energy transfer process to take place. From this latter state, an efficient singlet oxygen generation occurred, which was observed for the [Ru(dcbpy)_2_(mbpy-anth)]^2+^ complex,^20^ where mbpy is 4′-methyl-2,2′-bipyridine-4-carboxyamide and anth is an amidoanthracenyl moiety. These results indicated that the anthracenyl moiety could be employed to enhance singlet oxygen photoproduction and confer moderate DNA binding. In another step forward, we prepared a new ruthenium complex containing not only the anthracenyl bipyridine ligand but also a dppz ligand (dipyrido [3,2a:2,3-c] phenazine), [Ru(bpy)(dppz)(mbpy-anth)]^2+^,^18^ aiming to improve not only singlet oxygen photoproduction but also DNA binding. Actually, all those properties were enhanced, but we did not see any significant anticancer activity, which was only noticed after the incorporation of a biotin moiety to another bipyridine ligand ([Ru(bpy)(dppz)(mbpy-biotin)]^2+^).^11^ This latter multifunctionalized metal complex exhibited measurable anticancer activity, even though it showed a lower singlet oxygen photoproduction supporting a likely better uptake. The energy of the electronic states of these molecules can be tuned through the modulation of the ancillary ligand, which affects the energies of the π*(mbpy-anth) and dπ(Ru) orbitals and then modifying the pathways of deactivation involving photogeneration of ROS. By considering these issues and that multifunctionalization had improved only modestly the anticancer biological activity, we decided to investigate even simpler systems by altering the ancillary ligands such as [Ru(bpy)_2_(mbpy-ant)][PF_6_]_2_ (**GRBA**)^27,28^ and [Ru(phen)_2_(mbpy-anth)][PF_6_]_2_ (**GRPA**) complexes (Figure 1).

Interestingly, these reasonably simple metal complexes revealed remarkable properties, such as DNA interaction characteristics, photogeneration of reactive oxygen species, and antibacterial and anticancer activity, indicating that some simpler structures may work even better than some multifunctionalized compounds such as [Ru(dppz)(bpy-anth)]^2+^ or [Ru(dppz)(bpy-anth)(bpy-biot)]^2+^.

## Experimental Section

### Chemicals

Acetonitrile, ethanol, methanol, tetra-*N*-butylammonium perchlorate (PTBA), 2-aminoanthracene, 1,3-diisopropylcarbodiimide (DIC), 4,4′-dimethyl-2,2′-bipyridine, selenium dioxide, 1′-hydroxybenzotriazole hydrate (HOBT), calf thymus DNA, and *N*-methylmorpholine (NMM) were purchased from Sigma-Merck and ruthenium(III) chloride from Precious Metals Online (Australia). Methanol was treated with sodium sulfate, distilled, and stored in 4 Å molecular sieves. Dimethylformamide (Sigma/Merck) was distilled under low pressure and stored in 4 Å molecular sieves.

Biological assays employed tryptic soy broth (TSB) and tryptic soy agar (TSA) purchased from Himedia (India), buffer saline phosphate solution (PBS; pH 7.4), heat-inactivated fetal bovine serum (FBS), l-glutamine (200 mmol L^–1^), trypsin solution/EDTA (TE), and penicillin–streptomycin from Gibco Life Technologies. The culture medium Roswell Park Memorial Institute 1640 (RPMI 1640) and Dulbelcco’s modified Eagle medium (DMEM) were acquired from GE Hyclone. CellTiter 96 Aqueous MTS reagent powder was purchased from Promega Inc.

### Physical Measurements

Electronic spectra were done in a Cary 5000 UV–vis-NIR spectrophotometer (Agilent), using a 1 cm path length quartz cuvette. Fluorescence spectroscopy used a Quanta-Master QM-40 spectrofluorimeter (PTI) with a four-sided polished quartz cuvette. NMR spectra were obtained in deuterated solvents using a Bruker AVANCE DPX 300 spectrometer (300 MHz). Electrochemical measurements were carried out in an Epsilon potentiostat (Bioanalytical Systems Inc. (BAS)) using a single-compartment glass cell filled with deaerated acetonitrile (purged with argon) containing tetra-*N*-butylammonium perchlorate (0.1 mol L^–1^) as the supporting electrolyte and equipped with glassy carbon, platinum wire, and Ag/AgCl electrodes as working, auxiliary, and pseudoreference electrodes at 25 ± 0.2 °C. All electrochemical potentials described in this study were reported versus the Ag/AgCl electrode, which, under the given experimental conditions, were corrected using the standard value of ferrocenium/ferrocene (Fe^III/II^) redox pair (*E*_1/2_ = 0.410 V).^29^

### Synthesis

4′-Methyl-4-carboxylic acid −2,2′-bipyridine (bpy-COOH),^30^ 4′-methyl-*N*-(anthracene-2-il)-2,2′-bipyridine-4-carboxyamide (mbpy-anth), and **GRBA** ([Ru(bpy)_2_(mbpy-anth)](PF_6_)_2_)^27,28^ were prepared according to previous published procedures (all characterization can be checked in Figures S2, S3, and S5; solubility in water of 0.92 mg/mL and DTG: decomposition above 200 °C). [Ru(bpy)_2_Cl_2_] and [Ru(phen)_2_Cl_2_] were synthesized following the procedure reported in the literature.^31^

### [Ru(phen)_2_(mbpy-anth)](PF_6_)_2_ – (GRPA)

A suspension of [Ru(phen)_2_Cl_2_] (100.0 mg, 0.187 mmol) and mbpy-anth (75.0 mg, 0.192 mmol) in 30 mL ethanol/water (1:1) was heated and kept under reflux for 8 h. After this, the mixture was placed in a rotary evaporator under vacuum in order to concentrate up to 1/3 of its initial volume. Then, NH_4_PF_6_ was added and the mixture was stirred for 30 min and placed inside a refrigerator for 24 h. The orange-brown precipitate was then collected by filtration and washed with water and ether. Yield: 48%. ^1^H NMR (Figure S1 of the Supporting Information, 300 MHz, (CD_3_)_2_SO): δ 10, 93 (1H, s, 8), 8.88 (2H, s, 5 and 18), 8.83 (1H, d, 25), 8.71 (1H, d, 7), 8.64 (1H, s, 26) 8.59 (1H, s, 1), 8.53 (2H, m, 17 and 4), 8.49 (1H, s, 20), 8.38–8.33 (3H, m, 23, 28 and 30), 8.28 (1H, s, 16), 8.25 (1H, s, 11), 8.13–8.08 (2H, d, 12 and 15), 8.07–8.03 (4H, m, 10, 21, 27 and 33),7.97–7.88 (4H, m, 19, 24, 29 and 31), 7.80 (1H, d, 32), 7.71 (1H, d, 6), 7.53 (1H, d, 9), 7.51–7.45 (3H, m, 13, 14 and 22), 7.33 (1H, d, 2). FTIR: ν(C=O) amide 1635 cm^–1^. *E*_1/2_ = 1.28 V (vs Ag|AgCl). HR-MS: calculated 996.1588 and found 996.1633 [M+PF_6_]^+^. Anal. Calcd for C_50_H_35_ F_12_N_7_OP_2_Ru: C, 52.64; H, 3.09; N, 8.59; Found: C, 53.02; H, 3.12; N, 8.66. Solubility in water: 0.64 mg/mL. DTG: decomposition above 200 °C.

### DFT Calculation

Computational calculations were carried out at the National Center for High Performance Processing in São Paulo (CENAPAD SP) located at the State University of Campinas (UNICAMP). The software used was GaussView 5.0^32^ to generate the inputs and Gaussian09^33^ to perform the calculations on machines available in an IBM Power 750 Express Server environment. The geometries of the complexes were optimized using DFT (density functional theory), using the hybrid functional B3LYP.^34−36^ The LANL2DZ^37^ basis set was used to describe the ruthenium atom and 6- 31G(d)^38^ was used for the other atoms (C, H, O, N). Simulations involving the presence of the acetonitrile solvent were carried out using the polarized continuum solvation model (PCM).^39^ The theoretical electronic spectra were simulated using TD-DFT,^40^ also using the B3LYP functional and the mixed basis set mentioned above. The values of the percentage contributions of the orbitals were determined, and the electronic transitions were analyzed using the Chemissian 4.23 and GausssSum 3.0 software.

### Stability Measurements

Both metal compounds, **GRBA** and **GRPA**, were monitored using electronic spectroscopy and HPLC for up to 48 h in phosphate buffer pH 7.4 at 25 °C. In addition, the photostability of the metal compounds was also investigated and monitored for 270 min upon blue light irradiation. Chromatographic monitoring employed C-18 reverse phase column (5.0 μm, 4.6 mm × 150 mm, Waters) in an HPLC system (Shimadzu) equipped with a model LC-10AD pump and SPD-M20A photodiode detector. Samples of 10 and 20 μL were injected and run at a flow rate of 1 mL min^–1^, using a mixture of 15% methanol in water containing 0.1% NaTFA (sodium trifluoroacetate) at pH 3.5 as the mobile phase.

### Singlet Oxygen Measurements (^1^O_2_)

#### 1,3-Diphenylisobenzofuran

The reaction of ^1^O_2_ with 1,3-diphenylisobenzofuran (DPBF) was used to measure its quantum yield monitored by fluorescence. In all studies, a quartz fluorescence cuvette containing 2.5 mL of methanol with DPBF (20 μmol L^–1^) and **GRBA** (20 μmol L^–1^), **GRPA** (20 μmol L^–1^) or standard singlet oxygen photogenerators ([Ru(bpy)_3_]^2+^, methylene blue, rose Bengal) was used. This cuvette was irradiated with a blue (λ_max_ = 463 nm), green (λ_max_= 520 nm), or red (λ_max_= 631 nm) LED (all Basetech Conrand, 1.7 W), and the cuvette was placed back in the fluorimeter for measurement of the remaining DPBF fluorescence (λ_excitation_ = 410 nm, λ_max. emission_ = 479 nm). The consumption of DPBF was measured by the decrease in its fluorescence at 479 nm. The quantum yield of singlet oxygen production (Φ_Δ_) by the [Ru(bpy)_3_]^2+^ complex (Φ_Δ_ = 0.87),^41^ rose Bengal (Φ_Δ_ = 0.76), and methylene blue (Φ_Δ_ = 0.50),^42^ in saturated air-methanol, were taken as a reference for the blue, green and red light sources, respectively.

The oxygen singlet quantum yield was calculated according to the following equation:
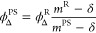
where ϕ_Δ_^PS^ is the quantum yield measured for **GRPA** and **GRBA** while ϕ_Δ_^PS^ is the quantum yield measured for the reference compound; m is the slope of the plot ln(*I*/*I*_0_) of DPBF (at 410 nm) vs irradiation time, and δ is the slope correction factor given by DPBF alone upon light irradiation.

#### SOSG

The reaction of ^1^O_2_ with a singlet oxygen sensor green probe (SOSG, ThermoFisher Scientific) was also used to measure its quantum yield as monitored by fluorescence. In these studies, a quartz fluorescence cuvette containing 2.5 mL of methanol or ultrapure water with SOSG (1 μmol L^–1^) and **GRBA** (10 μmol L^–1^) or **GRPA** (10 μmol L^–1^) or a standard singlet oxygen photogenerator ([Ru(bpy)_3_]^2+^ (10 μmol L^–1^) was used. This cuvette was irradiated with a blue LED (λ_max_ = 463 nm) and the cuvette was placed back in the fluorimeter for measurement of the SOSG fluorescence (λ_exc_ = 490 nm). In addition to this, a cuvette with only SOSG in methanol or water was light-irradiated, and its emission spectra as negative control were monitored. The singlet-oxygen quantum yield (Φ_Δ_) of the [Ru(bpy)_3_]^2+^ complex in water was previously reported as 0.41 and it was used for our relative quantum yield measurements.^41^

### Hydroxyl Radical Measurement (HO^**·**^)

This radical was detected using APF (aminophenyl fluorescein, ThermoFisher Scientific). The measurement of hydroxyl radicals was monitored by the increase in the emission band at *ca.* 515 nm as a function of the irradiation time using a blue LED (λ_max_ = 463 nm). All measurements were carried out using the metal complexes **GRPA** and **GRBA** at 10 μmol L^–1^ in 0.1 mol L^–1^ phosphate buffer pH 7.4 in the presence of APF (5 μmol L^–1^). Controls and selective suppressors were used to distinguish the reactive species generated, in particular, ^1^O_2_ and ^**·**^OH, for which sodium azide and D-mannitol were employed, respectively, both at a concentration of 10 mmol L^–1^.

### Superoxide Radical Measurements (O_2_^**·**–^)

The generation of superoxide radical (O_2_^**·**-^) mediated by l-glutathione (GSH) in an aqueous solution was carried out using nitrotetrazolium blue (NBT) method. This method is a conventional assay for measuring O_2_^**·**-^ radicals, which can reduce NBT producing blue formazan as a product with absorption around 560–590 nm. In this study, it was used a quartz cuvette containing 1.5 mL of phosphate buffer (PBS, 10 mmol L^–1^ phosphate at pH 7.4) with NBT (50 μmol L^–1^), GSH (1.5 mmol L^–1^), **GRBA** (5 μmol L^–1^), or **GRPA** (5 μmol L^–1^). These experiments were carried out in an aerobic environment, in the dark, and under blue LED irradiation (λmax = 463 nm) (Basetech Conrand, 1.7 W). The cuvette containing the mixture was placed back in the equipment to measure the absorbance of formazan formation (λ= ∼ 590 nm). Superoxide dismutase (SOD – 4 U/mL), a suppressor of superoxide anion radicals (O_2_^**·**-^), was also added as a control, in order to validate the production of this species, which can be noticed by suppression of the absorption band at ∼590 nm.

### DNA Measurements

#### Binding and Competition Assays

All DNA binding measurements employed 10 μmol L^–1^ of **GRPA** or **GRBA** in a conventional or fluorescence quartz cuvette containing 10 mmol L^–1^ of Tris-HCl pH 7.4 buffer. When using UV–vis electronic spectroscopy, titration was carried out by adding DNA into two identical quartz cuvettes, where one of which contained the metal complex and the other did not then all measurements were taken in a double beam arrangement using a Cary 5000 spectrophotometer. In this procedure, after each addition of calf thymus DNA (10 μmol L^–1^ in base pairs) into the cuvette, we waited for 5 min to collect its absorption or emission spectrum. There was also careful control to minimize changes in the total volume in the cuvette to avoid any significant dilution, which was always kept below 5% of the initial volume.

Investigations of the type of groove binding mode were carried out using methyl green and Hoechst agents, where the metal complexes were used as competitors, in 10 mmol L^–1^ Tris-HCl (pH 7.4) buffer at 25 °C. These measurements used Calf thymus DNA (CT-DNA) at 10 μmol L^–1^ along with 5 μmol L^–1^ of methyl green or Hoechst, which were titrated with metal complex **GRPA** or **GRBA** and monitored by fluorescence spectroscopy.

A competition assay using ethidium bromide (EtBr) was performed employing calf thymus DNA and ethidium bromide (1.5 μmol L^–1^), where an increasing amount of **GRPA** was added aiming to displace DNA-EtBr. This titration under competition was monitored by fluorescence at 600 nm. These data were fitted to a single binding equation using Prism 5 software (GraphPad), where an apparent dissociation constant (^APP^*K*_d_) was obtained. This value was applied in the competition binding equation below to estimate the *K*_d_ for **GRPA**.^43^ For this calculation, we used the dissociation constant for ethidium bromide (*K*_d_EtBr_ = 1.0 × 10^–7^) and concentration of [EtBr] = 1.5 μM. Thus, it is possible to calculate an estimated *K*_d_ value for **GRPA**.
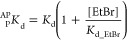


### DNA Cleavage Assay

This assay was carried out using the pBR322 plasmid along with **GRPA** and **GRBA** in 10 mmol L^–1^ tris buffer (pH 7.4). The metal complexes were mixed with DNA in increasing concentrations (0 to 10 μmol L^–1^) and incubated for 1 h at 25 °C either in the dark or upon light irradiation (blue, green, and red LEDs). All samples were applied into an agarose gel (0.8%), including a linear DNA ladder (1 kb, NEB) as a standard, which was separated by electrophoresis in TAE buffer. After this, agarose gel was incubated for 30 min with GelRed (Biotium, USA), and gel images were collected and analyzed in a Gel DocTM XR+ system (Biorad). In order to investigate the possible mechanism of DNA cleavage, reactive oxygen species scavenger assays were carried out with the addition of 20 mmol L^–1^ of selective reactive oxygen species quenchers along with the complexes under study, **GRBA** and **GRPA** (5 μmol L^–1^). The quencher reagents were pyruvate (for hydrogen peroxide disproportionation), histidine (singlet oxygen quencher), D-mannitol (hydroxyl radical quencher), and Tiron (superoxide anion radical quencher).

### Circular Dichroism Measurements

All circular dichroism measurements were carried out in 10 mmol L^–1^ tris-HCl buffer pH 7.4. Calf thymus DNA (350 μM in base pairs of a double-stranded DNA) was preincubated for 1 h at a fixed concentration of 100 μmol L^–1^ with the **GRBA** and **GRPA** metal complexes at various concentrations (2, 5, 7, 10, 15, 20, and 25 μmol L^–1^). These spectra were taken from 200 to 350 nm in a Jasco-815 instrument (Jasco), using a 1 cm path length quartz cuvette, 1 nm data density, 100 nm min^–1^ scanning speed, and 5 spectra accumulations. **GRBA** and **GRPA** metal complexes were also measured in the absence of DNA at their highest concentration.

### Partition Coefficient (Log *P*)

This measurement was done by following the well-established shake flask method with a nonmiscible *n*-octanol/water mixture. The concentrations of the **GRBA** and **GRPA** complexes (10 μM, in 0.25% DMSO/water) were first measured in water using a UV–vis spectrophotometer and then mixed with an equal volume of *n*-octanol. This suspension was stirred for 24 h in a sealed flask in the dark at 25 °C and centrifuged to achieve better phase separation. The aqueous layer was collected, and its spectrum was obtained. A similar measurement was carried out for Log *D*_7.4_, but the aqueous solution used was PBS buffer pH 7.4 instead. The concentration of these metal complexes in water or buffer was measured using standard curves (ABS vs concentration), where the concentration in the *n*-octanol layer was calculated by the difference from the concentration found in the aqueous layer and expressed as Log *P* as below.
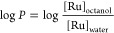

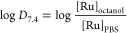


### Antimicrobial Assays

#### Microorganisms and Culture Conditions

In this study four bacterial strains were used: *Staphylococcus aureus* ATCC 25923 (methicillin-sensitive strain), *Staphylococcus aureus* ATCC 700698 (methicillin-resistant strain), *Staphylococcus epidermidis* ATCC 12228 and ATCC 35984 (isolated from a case of catheter sepsis, polysaccharide adhesin producer), *Escherichia coli* ATCC 11303, and *Pseudomonas aeruginosa* ATCC 27853, all from the American Type Culture Collection (ATCC). All strains were inoculated in TSA plates for 24 h at 37 °C and then individual colonies were subcultured into 10 mL of TSB and incubated for 24 h at 37 °C. Briefly, the bacterial culture was adjusted to a final concentration of 10^6^ colony-forming units (cfu)/mL.

#### Minimum Inhibitory Concentration and Minimum Bactericidal Concentration Determination

Bacterial susceptibility to the ruthenium complexes was determined using the minimum inhibitory concentration (MIC) and minimum bactericidal concentration (MBC) assays. The MIC and MBC values were measured by the microdilution method using microtitration in 96-well plates. Bacterial suspensions previously adjusted to 10^6^ cfu/mL were added into 96-well plates and then GRBA and GRPA at concentrations ranging from 1.9 to 250 μg/mL diluted in TSB containing 4% DMSO were added to the wells. The antibiotics, ampicillin, and tetracycline, were tested across the range of 0.04 to 1000 μg/mL and 0.39 to 100 μg/mL, respectively. These plates were irradiated with an array of 96 blue LEDs (10.8 J/cm^2^ of power) for 1 h or kept in the dark and then incubated at 37 °C for 24 h. After viewing the plates, the MIC values corresponded to the concentration of the compounds where there was no visible growth.

For measurement of MBC, an aliquot of 10 μL from each well with no visible growth was taken and used to inoculate TSA plates, incubated at 37 °C for 48 h. The MBC was considered to have the lowest concentration of ruthenium complex at which no colony growth was observed.

### Synergistic Effect

By employing the checkboard method, we evaluated the effects of ruthenium complexes, GRPA and GRBA, in combination with antibiotics.^44,45^ This assay uses multiple dilutions with two antibiotics at equivalent concentrations, below or above MICs for the microorganisms tested. For this study, two different combinations were evaluated, totaling four combinations, namely, GRPA + ampicillin, and GRPA + tetracycline; GRBA + ampicillin and GRBA + tetracycline. Thus, five wells of a 96-well flat-bottom polystyrene microplate were used for each combination tested, where 25 wells were used in a way that the final concentrations for each of the substances were at the MIC, 1/2 MIC, 1/4 MIC, 1/8 MIC, and 1/16 MIC. Immediately after assembling the plates, they were incubated at 37 °C for 24 h, and new MIC was measured in combination. The OD was measured at a wavelength of 620 nm in a microplate reader (Spectramax). These data were interpreted by determining the fractional inhibitory concentration index (FICI), obtained by adding up the values of the fractional inhibitory concentration (FIC) of each compound used in the combination, according to the equation below:
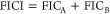
FIC_A_ = [MIC of the metal complex in combination/MIC of the individual complex]; FIC_B_ = [MIC of the antibiotic in combination/MIC of the individual antibiotic].

According to the values obtained, a fractional inhibitory concentration index (FICI) was calculated for each combination in order to assess the type of interaction between the drugs. Thus, this interaction was considered synergistic (FICI ≤ 0.5), indifferent (0.5 < FICI ≤ 4), or antagonistic (FICI > 4).^46^

### Cytotoxic Assay

All human cell lineages, MDA-MB-231 (triple-negative human breast cancer), A549 (human lung cancer), and MRC-5 (healthy human lung fibroblast), were grown in modified Dulbecco cell culture medium (DMEM, *Dulbecco’s Modified Eagle Medium*), supplemented with 10% of fetal bovine serum (FBS). Cell lineage A2780 (human ovarian cancer cells) was grown in the RPMI 1640 medium (*Roswell Park Memorial Institute*), supplemented with fetal bovine serum at 10% (FBS). All cells were kept in a CO_2_ (5%) incubator at 37 °C.

### Determination of Cellular Viability

The cellular viability tested with the compounds was determined using the MTT (3-(4,5-dimethylthiazol-2-yl)-2,5-diphenyltetrazolium bromide) colorimetric method.^47^ Initially, the cells were trypsinized to count and adjust cell concentration, then seeded in 96-well culture plates (1.5 × 10^4^ cells/well) and subsequently incubated in an incubator (at 37 °C and 5% CO_2_) for 24 h for cell adhesion. After this period, compounds were added into the wells at different concentrations (0.012 to 50 μmol L^–1^), containing a final concentration of 0.5% of DMSO, and the plates were kept in the incubator again for a further 48 h. The culture medium was then removed from the plates and 50 μL of MTT (1.0 mg mL^–1^ in PBS) was added to each well, which was then incubated in the oven for 3 h.

For the light irradiation experiments, 96-well plates were initially seeded and incubated for 24 h. The compounds were added and the plate was kept in the incubator for another 24 h (37 °C and 5% CO_2_). The culture medium was replaced by fresh medium without phenol red, and the plate was irradiated for 10 min and kept in the incubator for a further 24 h ((λ_irrad_ = 460–465 nm, 18 mW cm^–2^, 10.8 J cm^–2^). After this period, the culture medium was removed, 50 μL of MTT (1.0 mg mL^–1^ in PBS) was added to each well, and the plate was incubated for a period of 3 h.

In both experiments, the formazan crystals formed were solubilized by adding 150 μL of DMSO, and the absorbance was recorded at 540 nm on a Synergy/H1-Biotek spectrophotometer/fluorimeter. The negative control cells were also cultivated with medium containing 0.5% DMSO. The IC_50_ values were calculated using GraphPad Prism 8 software.

## Results and Discussion

### Characterization of the Ruthenium Complexes

The modified ligand, mbpy-anth,^30^ was obtained via carbodiimide activation, while **GRBA** ([Ru(bpy)_2_(mbpy-anth)](PF_6_)_2_) and **GRPA** ([Ru(phen)_2_(mbpy-anth)](PF_6_)_2_) were prepared according to the literature^18,20^ and isolated as PF_6_^–^ salt with yields of 52 and 48%, respectively. Spectroscopic characterization of **GRBA** was already reported^27^ and has been used here for quality and comparison purposes. These compounds had their constitution and purity assessed by NMR spectroscopy (Figures S1 and S2), electronic absorption (UV–vis), FTIR technique (Figures S3–S5), and elemental analysis. For **GRPA**, the full assignment of ^1^H and ^13^C NMR signals was provided with the aid of two-dimensional techniques ^1^H–^1^H correlated spectroscopy (COSY)). ^1^H NMR (Figure S1) of **GRPA** showed signals at 2.43 and 10.94 ppm, attributed to the hydrogens of the methyl and amide groups found in the bipyridine modified with the anthracenyl moiety, respectively. In addition, the FTIR spectrum showed also a characteristic stretching band of C=O of amides at 1635 cm^–1^ and a broad strong band at 840 cm^–1^ due to the stretching vibration mode of the PF_6_^–^ counteranion.

### Electrochemical Studies

Electrochemical data for the two metal complexes in acetonitrile are illustrated in Figures S7 and S8 and data summarized in Table 1. The half-wave potentials (*E*_1/2_) assigned to the Ru^III/II^ redox pair of **GRPA** and **GRBA** were determined by cyclic voltammetry at 1.28 and 1.16 V vs. Ag/AgCl, respectively (Figures S7 and S8). This cathodic potential shift for **GRPA** could be ascribed to a greater π-back-bonding effect afforded by the *o*-phenanthroline ligand. A noticeable difference between these voltammograms is an additional oxidation process at *ca.* 1.21 V for **GRPA**. There is a similar process previously assigned to the anthracene oxidation for [Ru(bpy)(dppz)(mbpy-anth)]^2+^ complex (where dppz is dipyrido[3,2-a:2′,3′-c]phenazine).^18^ The *o*-phenanthroline ligand has an essential structural role in the metal complex by increasing interaction between the π electron density of the anthracene and the amide bridge as similarly noticed when dppz was used instead. Actually, this result is in agreement with DFT calculations, where both metal complexes showed the lowest unoccupied molecular orbital (LUMO) mainly on the bipyridine (bpy) moiety (Figures S9 and S10). The highest occupied molecular orbital (HOMO) for **GRBA** was found on the anthracenyl moiety, while for **GRPA,** it was found on the anthracenyl, including the amide bridge. Besides that, three redox waves are observed at negative potentials, which are due to the reduction of the polypyridinic ligands.

**Table 1:** Absorption Spectra, Extinction Coefficients, and Electrochemical Data for **GRPA** and **GRBA**

complex	λ_abs_/nm (ε × 10^4^/ mol L^–1^ cm^–1^)	*E*_ox_/V	*E*_red_/V
**GRPA**	244 (5.50), 261 (9.19), 290 (3.71), 388 (1.07), 455 (1.20), 580 (0.17)	1.21, 1.28	–1.11, −1.41, −1.88
**GRBA**	245 (2.99), 288 (8.60), 422 (1.79) 459 (1.95)	1.16	–1.44, −1.65, −1.91
[Ru(bpy)(dppz)(mbpy-anth)]^2+^^a^	257 (52), 276 (54), 360 (1.8)460 (2.7)	1.30, 1.33	–0.96, −1.61, −1.78

a From ref (18). Spectroscopy data in methanol and electrochemistry in acetonitrile at 25 °C.

### Electronic Spectroscopy

**Figure 2:**
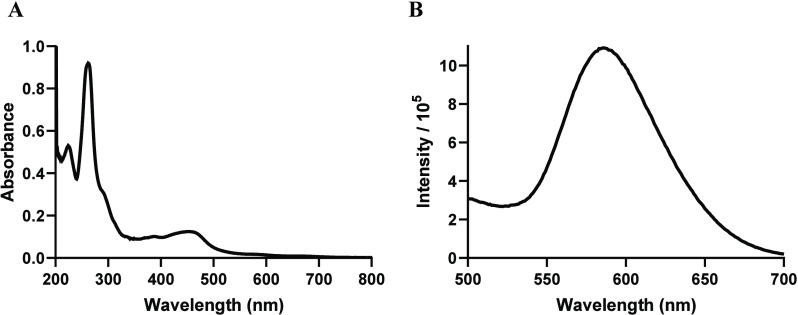
Electronic absorption (A) and emission (B) spectra of **GRPA** in methanol (2 × 10^–5^ mol L^–1^) at 25 °C (λ_exc_ at 446 nm).

The absorption spectrum of **GRPA** was recorded in the methanol solution (Figure 2), where its absorption band maxima (λ_abs_) and molar extinction coefficients (ε) are listed in Table 1. These data for **GRBA** are shown in Figure S5A. As commonly described for polypyridine metal complexes, there are typical π–π* transitions relative to phen and bpy ligands in the UV region. In addition, a broad band of low energy absorption can be seen assigned to metal-to-ligand Ru(dπ) → Lπ*) charge transfer (MLCT) involving bpy or phen as π* acceptor orbitals. A comparison of the spectroscopic features of **GRPA** and **GRBA** demonstrates the impact of the phen once replacing bpy. In the UV absorption band, **GRPA** is shifted hypsochromically by 27 nm relative to **GRBA**, which can be rationalized by taking into account the extended π-electron system of phen that leads to a greater energy gap for the π → π* transition. Furthermore, there is a structured absorption in the range of 330–390 nm for **GRPA**, which is typical of anthracene. On the other hand, **GRBA** does not show this vibronic absorption profile. These observations suggest that an interaction between 2-anthracenyl and bpy unit is slightly different for both metal complexes, which is modulated by the other ancillary ligands. In the lower energy region of the spectrum, the MLCT band of **GRPA** is slightly broader than that observed for **GRBA**, which probably results from an overlap of Ru(dπ) → phen/bpy(π*) transitions. Their maximum values for Ru(dπ) → bpy(π*) transitions for **GRPA** and **GRBA**, in methanol, were at 455 and 459 nm, respectively. Moreover, a weaker band for **GRPA** is also observed at lower energy at *ca.* 580 nm, which has been assigned to an intraligand charge transfer (ILCT) transition from the HOMO (π) residing on the fragment amide-anthracenyl to the LUMO (π*) localized on the modified 2,2′-bipyridyl fragment, as predicted from TD-DFT calculations (Table S1).

Aiming to shed some light on the differences between these two metal complexes, including their spectroscopic features, we made some comparisons using the starting compounds. There are two characteristic spectroscopic profiles for 2-aminoanthracene (anth-NH_2_): one broad and structureless band with a maximum around 400 nm and another one at higher energy formed by vibronic transition bands (320–350 nm) (Figure S6). These two low-lying excited states exhibit π–π* character and are denoted as ^1^L_a_ (B_2u_) and ^1^L_b_ (B_3u_), respectively, according to Platt’s nomenclature.^48^ The substitution of a hydrogen atom by an electron-acceptor bipyridine group on the amino group of 2-aminoanthracene ligand reduces π conjugation between the amino and anthracene groups, causing an opposite effect on the two absorption bands. The first band (^1^L_a_) shifts to higher energy while the second one to lower energy. Additionally, the absorption of the vibrational band (^1^L_b_) increases, meaning that it becomes optically allowed. Indeed, the bichromophoric behavior of **GRBA** was already reported,^27,28^ showing independent characteristic profiles of ruthenium-bipyridine and anthracene. However, **GRPA** exhibits characteristic profiles of the ruthenium-bipyridine and amido-anthracene moieties due to a greater π-acceptor effect of the *o*-phenanthroline ligand, where spectroscopic and electrochemical data come to support this phenomenon. In this case, vibrational bands are more intense allowing the amide bridge to donate electrons to bipyridine, thereby facilitating oxidation of the anthracene fragment.

The luminescence spectrum of **GRPA,** excited at 450 nm, exhibited a broad and structureless emission band with a maximum at 601 nm, in methanol (Figure 2B). This emission profile is characteristic of ^3^MLCT due to its similarity to **GRBA**, which may suggest that emission originates from the MLCT involving bipyridine modified with anthracene. Table 2 shows the maximum emission values and emission quantum yield for **GRPA** and **GRBA** in different solvents.

**Table 2:** Emission Quantum Yield (Φ_em_), Maximum Emission Values (λ_max,_ nm), and Singlet Oxygen (Φ_Δ_) Quantum Yield for **GRPA** and **GRBA**

complex	methanol			H_2_O	ethanol	DMF	dichloromethane
	Φ_em_ (λ_max_)	Φ_Δ_^a^	Φ_Δ_^b^	Φ_Δ_^b^	Φ_em_ (λ_max_)	Φ_em_ (λ_max_)	Φ_em_ (λ_max_)
**GRPA**	0.039 (601)	0.95	0.94	0.96	0.046 (606)	0.078 (635)	0.093 (591)
**GRBA**	0.033 (603)	0.45	0.52	0.52	0.040 (604)	0.069 (623)	0.086 (588)

a ^1^O_2_ measured using DPBF probe.

b ^1^O_2_ measured using SOSG probe.

All emission bands shifted toward longer wavelengths in more polar solvents, while quantum yield decreased. There is a moderate change in quantum yields from 3.0 up to 9.0% in all of these solvents. This can be explained once the excited state is stabilized in polar solvents, however, there is an increase in energy transfer to nonemissive states ^3^IL/^3^ILCT, thereby reducing the quantum yield of emission as described.

**Figure 3:**
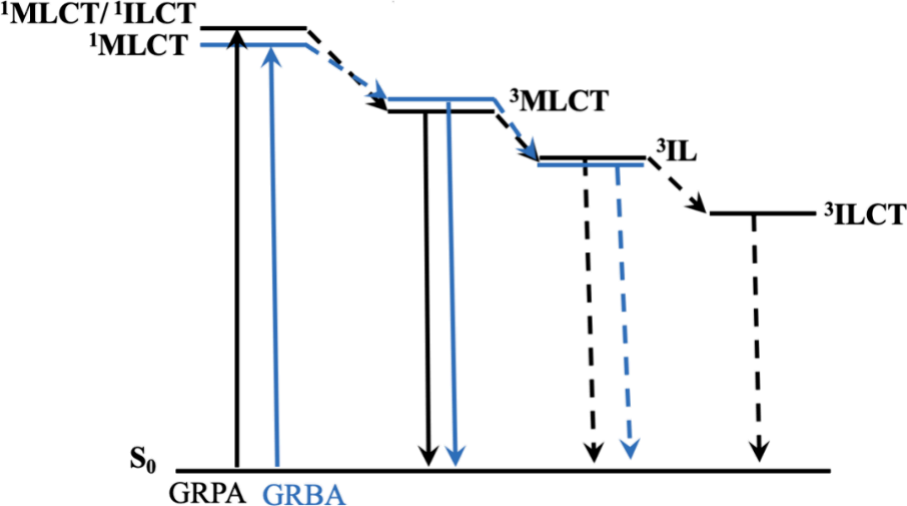
Schematic of the energy levels for **GRPA** (black line) and **GRBA** (blue line).

A simplified energy diagram is shown in Figure 3 for these metal complexes. There is an emission process occurring with both metal complexes from the low-lying ^3^MLCT state of mbpy-anth. The low emission process observed for **GRBA** is attributed to the energy transfer to the lower ^3^IL (^3^anth).^27^ Meanwhile, **GRPA** presents low-lying nonemissive ^3^IL and ^3^ILCT states. For **GRPA**, this ^3^ILCT would also sensitize the conversion of ^3^O_2_ to ^1^O_2_, which would make us expect an enhanced process.

### Thermal and Photochemical Stability

These metal compounds were subjected to biological assays with long incubation times and also light irradiation as further described. Based on this, it was important to guarantee that they were indeed stable under these conditions. To investigate this, first of all, electronic spectroscopy was employed by monitoring changes, if any, for **GRBA** and **GRPA** during 48 h of incubation in 10 mmol L^–1^ of phosphate buffer pH 7.4 at 37 °C. Additionally, samples of these compounds were also irradiated with blue light for up to 270 min and monitored by electronic spectroscopy. Our results did not show any evidence of spectroscopic changes indicating no decomposition of these compounds under these conditions (Figure S11). Besides this, we also employed high-performance liquid chromatography (HPLC) to monitor the integrity of these metal compounds for up to 48 h in the dark and also for 1h irradiated with blue light. A sharp peak with a retention time of 3.61 min was observed for **GRBA** with consistent electronic spectra, which did not change even after 48 h of incubation in 10 mmol L^–1^ phosphate buffer pH 7.4 (Figure S12) neither with blue light (Figure S13). A similar behavior was also seen for **GRPA**, which was eluted with a retention time of 1.8 min without changes during 48 h (Figure S14), while no changes were noticed with blue light (Figure S15). Altogether, these results supported the stability of these compounds, allowing them to be further explored for biological purposes.

### Singlet Oxygen Production (^1^O_2_)

The quantum yield of singlet oxygen generation for anthracene-containing ruthenium complexes was measured in methanol using the fluorescent probe 1,3-diphenylisobenzofuran (DPBF) under light irradiation.^11^ There are interesting cases of expressive enhancement of the photoproduction of singlet oxygen upon conjugation of an anthracenyl group to the metal complexes.^18,20^ The photoproduction of singlet oxygen mediated by **GRPA** and **GRBA** was analyzed under irradiation with blue (λ_max_ = 463 nm), green (λ_max_ = 520 nm), and red (λ_max_ = 632 nm) light.

Our results showed significantly high values for the generation of singlet oxygen for both metal complexes (Figure S16). **GRPA** showed a superior performance, with a very high quantum yield value of Φ_Δ_= 0.95. This can be attributed to the participation of the ^3^ILCT and ^3^anth (anthracenyl moiety) triplet state in the energy transfer processes (Figure 3). If the visible light-absorbing MLCT transition is excited, intersystem crossing to the ^3^MLCT state will take place. Then, the ^3^MLCT state may act as an efficient energy transfer channel to excite ^3^O_2_ to ^1^O_2_ due to the proximity of their energy levels. The differences in quantum yields between **GRPA** (Φ_Δ_= 0.95) and **GRBA** (Φ_Δ_= 0.45) can be attributed to the presence of a lower energy ^3^ILCT state found in the **GRPA** complex.^49^

In addition, it was observed that **GRPA** performed also better under irradiation with green (Φ_Δ_= 0.22) and red (Φ_Δ_= 0.11) light if compared to **GRBA**, which did not show measurable quantum yield under those conditions. These results indicate the potential of the metal complexes as efficient systems for generating singlet oxygen species, highlighting the importance of the anthracenyl group along with phenanthroline ligands improving the overall photosensitivity of the ruthenium complexes. Beyond this, other modifications in the auxiliary ligands should be carefully considered as well. An analogous compound of **GRBA** containing dicarboxylic-2,2′-bipyridines ligands instead showed a higher quantum yield of singlet oxygen with blue light (Φ_Δ_ = 0.76).^20^ However, these carboxylic substituents altered quite expressively the solubility of the metal complex, making them harder to carry out any further investigations.

**Figure 4:**
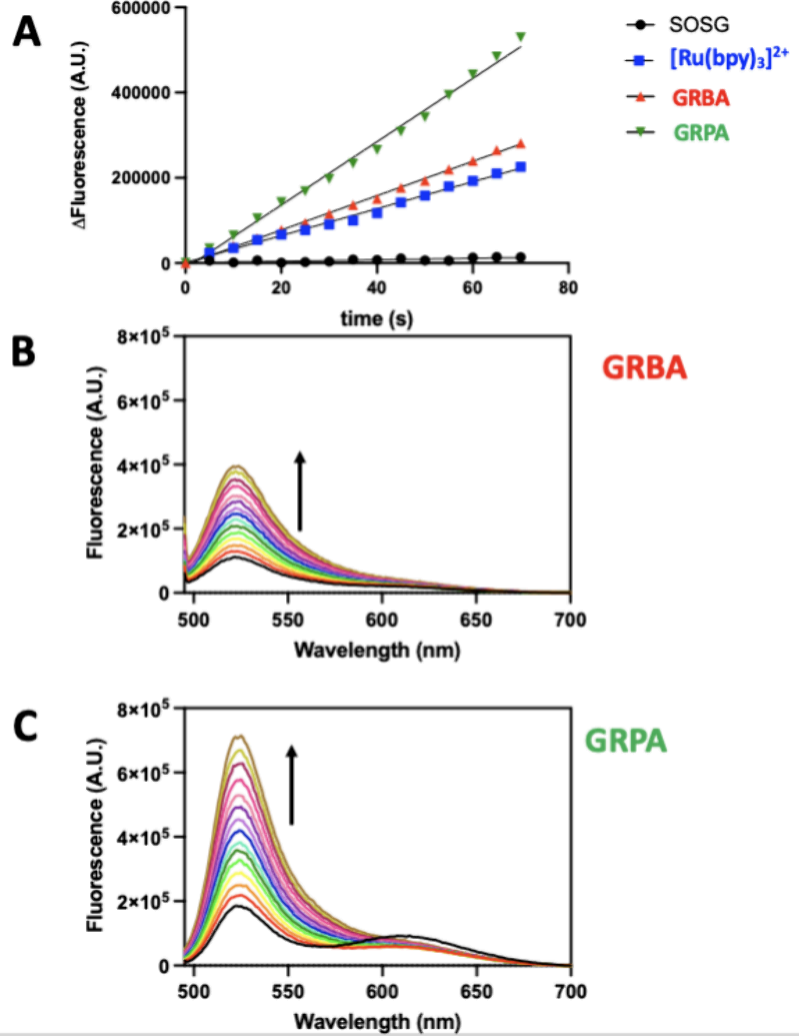
Measurement of the production of singlet oxygen upon blue light irradiation using SOSG probe (1 μmol L^–1^) in ultrapure water (λ_exc_ at 490 nm). Panel A shows a linear change of fluorescence during light irradiation in water for SOSG (alone, black circle), **GRPA** (green inverted triangle), **GRBA** (red triangle) and [Ru(bpy)_3_]^2+^ (blue square). Panel B and C show the raw emission spectra for SOSG with **GRPA** (10 μmol L^–1^) and **GRBA** (10 μmol L^–1^) during blue light irradiation.

Despite the common use of DPBF as a sensitivity oxygen singlet probe, there are reports pointing out its lack of selectivity,^50^ where superoxide could also contribute and be mistakenly detected.^51^ Aiming to prevent this eventual issue, we also measured singlet oxygen species by employing a commercial highly selective probe known as singlet oxygen sensor green (SOSG).^52^ In addition to a superior selectivity, this probe can also be used in an aqueous medium enabling us to measure a relative quantum yield in such conditions. Thus, we carried out measurements in methanol to compare with DPBF, and also in ultrapure water (at *ca.* pH 7.0). We still noticed an expressively strong photoproduction of singlet oxygen by **GRPA** in comparison to **GRBA** either in water (Figure 4) or methanol (Figure S17), Table 2. Notably, **GRPA** (Φ_Δ_ = 0.96) showed almost double the singlet oxygen quantum yield reported for [Ru(bpy)_3_]^2+^ in water (Φ_Δ_ = 0.41),^41^ supporting its enhanced efficiency.

### Hydroxyl Radical Production

**Figure 5:**
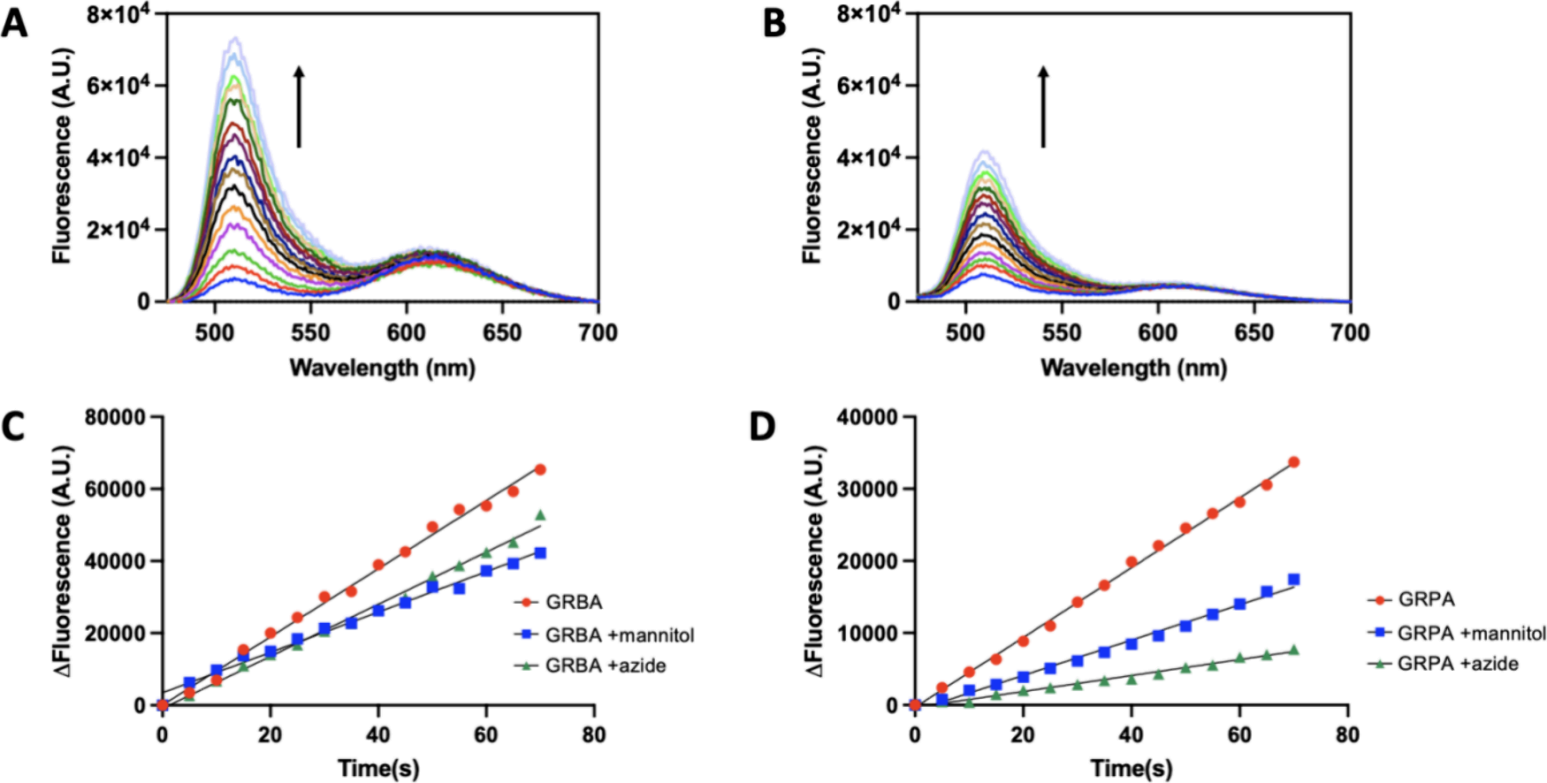
Measurement of the production of hydroxyl radical upon blue light irradiation using APF probe (5 μmol L^–1^) in 100 mmol L^–1^ phosphate buffer pH 7.4 (λ_exc_ at 463 nm). Panels A and B show emission spectra for APF with **GRBA** (10 μmol L^–1^) and **GRPA** (10 μmol L^–1^) during blue light irradiation. Panels C and D show a linear change of fluorescence during light irradiation in phosphate buffer for APF with **GRPA** and **GRBA,** and also with the addition of mannitol (10 mmol L^–1^) or sodium azide (10 mmol L^–1^), respectively.

Hydroxyl radicals (HO^**·**^) are the strongest oxidizing reactive species reported (*E*°’(HO^**·**^/H_2_O) = 2.34 V),^53,54^ which can be detected using a probe known as APF (3′-(*p*-aminophenyl)fluorescein). This probe has been considered very selective to this radical, but we and others noticed that singlet oxygen could still be an issue.^20,54^ Here, we monitored the relative production of this radical upon blue light irradiation in methanol, where scavengers for HO^**·**^ (mannitol) and ^1^O_2_ (sodium azide) were also employed (Figure 5). Interestingly, **GRBA** showed *ca.* 2-fold faster photoproduction of hydroxyl radical than **GRPA**, while this latter was more sensitive to sodium azide as expected based on its stronger photoproduction of singlet oxygen. This radical may also emerge from superoxide, which was further investigated.

### Superoxide Production

Another important reactive oxygen species is the superoxide radical (O_2_^**·**–^), which can be produced through photochemical processes. This species can be investigated using selective probes such as nitrotetrazolium blue (NBT).^55^ In vitro assays were carried out to analyze O_2_^**·**–^ production in a mixture containing NBT, ruthenium complex, and reduced glutathione (GSH). This latter compound is a common reducing agent found in millimolar concentrations inside cells, which could support electron transfer processes. These experiments were conducted in 10 mmol L^–1^ of phosphate buffer pH 7.4 for 100 min at 25 °C. Some control samples were also prepared, such as a combination of the metal complexes with NBT and another one combining only GSH with NBT, which were light-irradiated or monitored in the dark (Figure S18). Indeed, there are neither spectroscopic changes in all control samples investigated nor an electronic band at *ca.* 590 nm, even when metal complexes were combined with GSH and NBT in the dark, indicating the lack of any generation of superoxide species.

**Figure 6:**
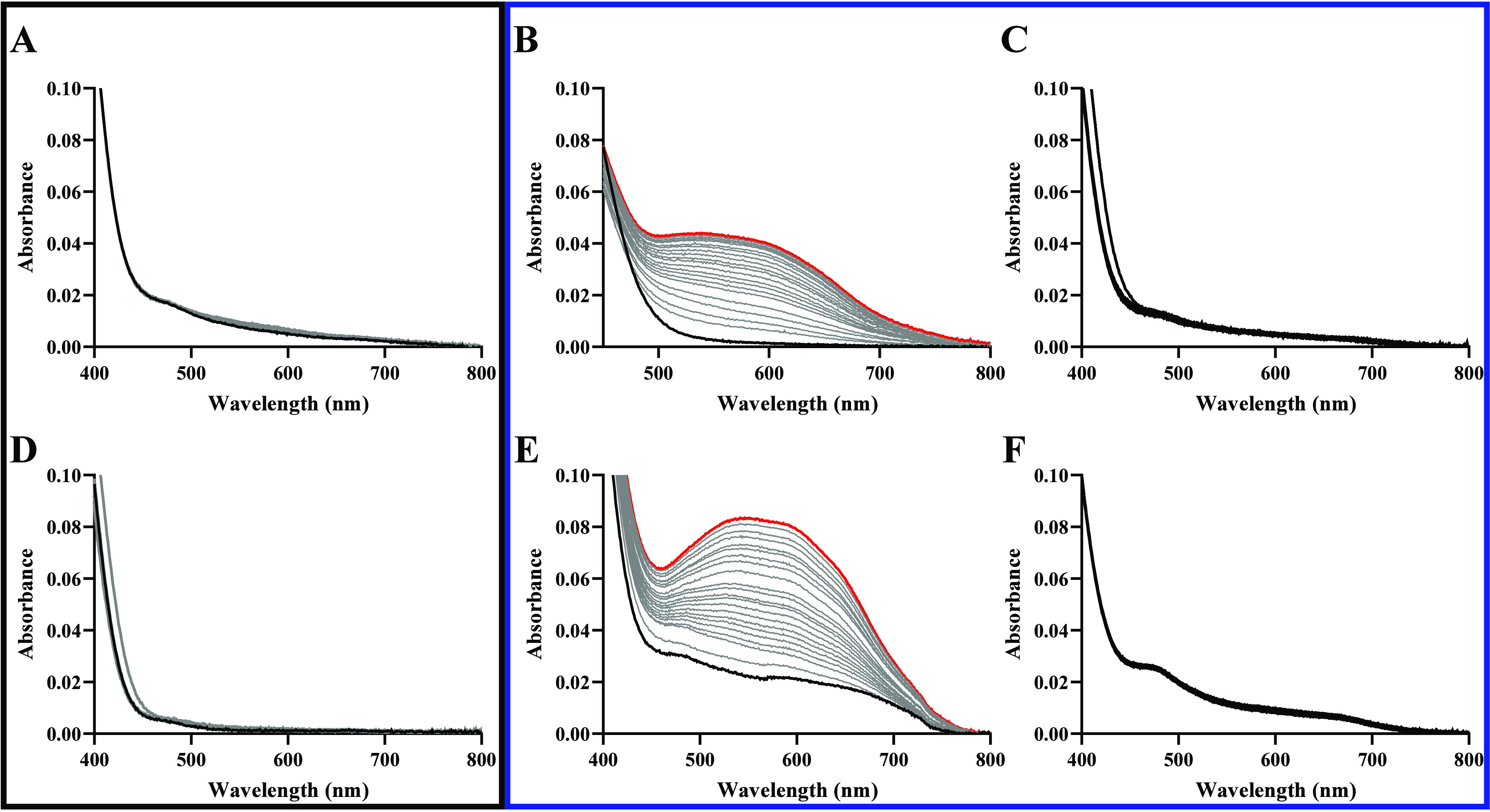
Superoxide detection using NBT (50 μmol L^–1^), GSH (1.5 mmol L^–1^) and **GRPA** (5 μmol L^–1^), monitored for 100 min in the dark (A), with blue light irradiation for 100 min (B) and in the presence of SOD with blue light irradiation (C); similarly, for **GRBA** (5 μmol L^–1^) in the dark (D), with blue light irradiation (E) and in the presence of SOD with blue light irradiation (F). All reaction were carried out at 25 °C.

However, if blue light is employed, along with GSH, there is a remarkable change in the electronic spectrum with a broad and intense band seeing at *ca.* 590 nm (Figure 6). This profile is typical of the reduction of NBT promoted by the superoxide reaction with the formation of formazan, which indicates that under blue light irradiation, this radical is indeed produced. When comparing side by side the generation of superoxide radicals promoted by both metal complexes, we observed that **GRBA** produced this species *ca.* 1.8 times faster than **GRPA** (Figure S18). In the cuvette, a color change is visually noticed going from yellow to a blue/purple solution. This is consistent with the formation of formazan with an electronic band with a maximum at *ca.* 590 nm. It is important to mention that this change promoted by the metal complex is not seen in the absence of GSH and light (Figure S18). Based on that, it is clear that both stimuli are essential to ensure superoxide production. Nevertheless, to further validate that superoxide is indeed being produced, we added the superoxide dismutase enzyme (SOD). This enzyme can very quickly decompose superoxide in oxygen and hydrogen peroxide, working as an efficient scavenging agent. By adding this enzyme to the reaction mixture containing all other components (metal complexes, NBT, and GSH) and submitting it to irradiation with blue light, there were no significant changes at 590 nm (Figure 6), supporting our previous observations. This photoreaction process may require GSH to provide electrons for a cyclic production of superoxide. Notably, **GRBA** was the most efficient in the photogeneration of superoxide radicals through a mechanism of type I (Figure 3). This may be very relevant for applications related to photodynamic therapy and other areas of investigations, where controlled production of this strongly reactive radical is desired.

In fact, while the less positive oxidation potential of **GRBA** promotes more effective electron transfer donation, the low triplet energy of **GRPA** (^3^ILCT) facilitates oxygen quenching by energy transfer.

### Interaction with DNA

**Figure 7:**
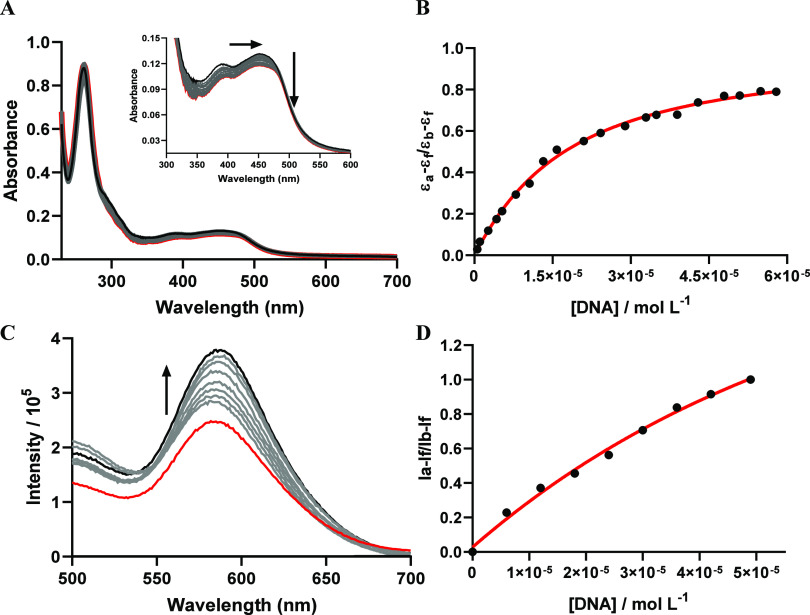
DNA binding measurements. Panels show the titration of **GRPA** with calf thymus DNA monitored by UV–vis absorption electronic spectra (A), plot of ε_a_ – ε_f_ /ε_b_ – ε_f_ vs [DNA] (B), luminescence with excitation at 450 nm (C) and plot of (*I*_a_ – *I*_f_)/(*I*_b_ – *I*_f_) vs [DNA]) (D) in 0.1 mol L^–1^ Tris-HCl pH 7.4 at 25 °C.

In order to evaluate the strength of the interaction of the **GRBA** and **GRPA** with calf thymus DNA (CT DNA), we measured this binding by UV–vis and luminescence spectroscopies.^56^ By titrating CT-DNA into a solution containing **GRPA**, we observed changes in both electronic absorption and emission spectra (Figure 7). We noticed a maximum change in the electronic transition band at 450 nm of only 2 nm and a hypochromic effect of 11.3% involving intraligand transition bands, while a hypochromic of 18.15% for MLCT band was seen. Based on these measurements, we calculated a binding affinity constant (*K*_b_) for the interaction of **GRPA** with CT-DNA which was of 4.51 (±0.2) × 10^4^ at 25 °C.

Even though the profile cannot ensure the mode of interaction of the metal complex with DNA, the extension of the hypochromism might suggest a possible intercalative interaction of that metal complex.^57^ Nonetheless, these observations indicate clearly an interaction of the metal complex with this macromolecule. The binding affinity measured for **GRPA** is still moderate but consistent with *K*_b_ values found in the literature for other similar metal compounds with intercalative behavior, such as [Ru(phen)(dicnq)_2_]^2+^ (*K*_b_ = 3.30 × 10^4^)^58^ and [Ru(phen)_3_]^2+^ (*K*_b_ = 2.80 × 10^4^).^59^ This value is still moderate if compared to other metal compounds with undisputable mechanisms of interaction through intercalation, for example, [Ru(bpy)_2_(dppz)]^2+^ (*K*_b_ = 3.2 × 10^6^).^60^ On the other hand, for **GRBA**, no significant changes were observed in the absorption electronic spectrum, indicating that there is likely no relevant interaction of the metal complex with the DNA. This behavior may be due to structural differences between **GRBA** and **GRPA**, where the latter containing *o*-phenanthroline ligands have a larger aromatic planar structure available with higher hydrophobicity as well. These features could improve considerably the metal complex affinity to DNA.^61^ It is important to mention that an analogous metal complex, containing two dicarboxylic-2,2′-bipyridine ligands instead of 2,2′-bipyridine as in **GRBA**, showed measurable DNA binding (*K*_b_ = 5 × 10^4^)^20^ in very close proximity to **GRPA**. This result suggests that DNA binding can be very sensitive to even minor modifications in the auxiliary ligand, where smaller substituent groups can influence even if incorporating negative charges.

In addition to UV–vis absorption measurements, luminescence studies also confirmed an efficient binding of **GRPA** to DNA. This technique can be even more sensitive to such interactions, assisting in validating this phenomenon. Therefore, titrating CT-DNA into a cuvette containing **GRPA** caused a consistent increase in light emission, suggesting the formation of a metal complex-DNA interaction (Figure 7). These data were further analyzed using a single binding equation and provided a binding affinity constant of 1.57 (±0.15) × 10^4^ (or *K*_d_ = 64 ± 6 μmol L^–1^) which is in close agreement with the UV–vis absorption binding data. This behavior with DNA interaction has been widely described for other similar intercalator molecules,^11,18,20^ where binding to DNA occurs involving hydrophobic microenvironment patches, protecting the metal complex from the aqueous medium. When the metal complex is intercalated, it is stabilized by π–π stacking interactions, inducing structural changes in the DNA. In this way, this possible mode of interaction ends up causing structural changes in the DNA itself, conferring stability, rigidity, and unwinding of the double helix structure.^62^

For **GRBA**, there were no changes in the spectroscopic profiles, indicating that there is possibly no interaction with DNA (Figure S19). Structurally comparing **GRBA** with **GRPA**, there is a larger planar structure due to the *o*-phenanthroline ligand in **GRPA** that provides a higher aromatic planarity area and hydrophobicity. This might be a key to conferring a considerable *K*_b_ value allowing significant affinity to DNA.^61^

Furthermore, other studies with DNA have been done using a competition experiment with ethidium bromide (EtBr), a well-known fluorescent intercalator. In a competition experiment, EtBr which shows strong fluorescence upon binding to DNA can be displaced by the addition of a competitor, leading to a decrease in its own emission. This strategy provides some evidence of the intercalative mode of interaction, as well as the binding strength of the compound toward DNA.^63^ In the case of the **GRPA**, we explored this interaction using a competition measurement with 1.5 μmol L^–1^ of EtBr and 10 μmol L^–1^ of DNA (in base pairs) followed by gradual additions of **GRPA**. After each addition of the metal complex, we observed a consistent decrease in the emission band, suggesting that a competition process was taking place involving **GRPA** and EtBr for binding to DNA.^62^ However, we noticed that even after adding *ca.* 11 μmol L^–1^ of the metal complex, there was no complete decrease in the intensity of the emission band. This behavior can be attributed to the intrinsic emission of the metal complex itself once bound to DNA, which was described earlier (Figure S20).^11,18^ Nevertheless, we estimated the affinity of **GRPA** to DNA in this competition experiment, which indicated a dissociation constant (*K*_d_) of 4.7 ± 0.2 μmol L^–1^. This value is not so close to the one measured by direct titration of DNA, which could indicate distinct sites of interaction being EtBr in a high affinity one for **GRPA**.

Beyond this type of binding, ruthenium(II) polypyridine complexes can also exhibit a preference for DNA grooves. To access this information, we explored another competitive measurement using methyl green and Hoechst fluorescent probes since they are known to interact selectively with the major and minor groove of the DNA molecule, respectively. By using methyl green, we noticed only minor changes upon titration with both metal complexes (Figure S21), suggesting that there is no significant preference for the major groove. On the other hand, a competition experiment with Hoechst showed upon the addition of **GRBA** or **GRPA** that there was a strong decrease in the luminescence intensity (Figure S22). These results indicated both metal complexes exhibit a higher preference for interaction with the minor groove of the DNA. These titrations provided also an estimative for the strength of these interactions (Hoechst, *K*_d_ = 0.140 μmol L^–1^),^18^ where *K*_d_ of 6.3 and 9.1 μmol L^–1^ were measured for **GRBA** and **GRPA**, respectively. Interestingly, these titration curves were fit to an equation with the Hill slope, indicating some possible synergistic interactions. This experiment is quite interesting considering that in previous attempts, we did not notice any significant interaction of **GRBA** with DNA and maybe hindered by minor electronic disturbance upon binding.

### Circular Dichroism

**Figure 8:**
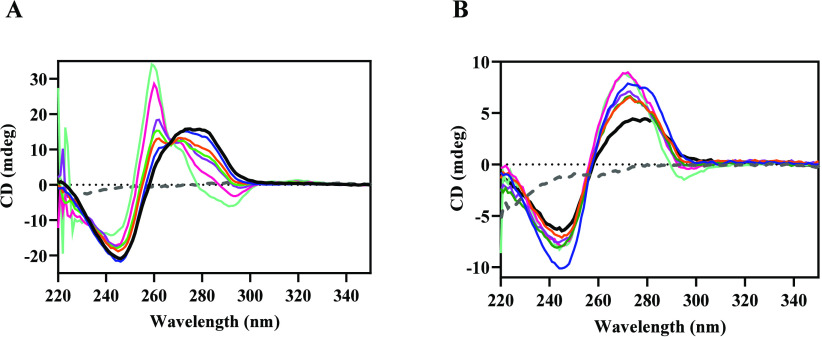
Interaction of the metal complexes with DNA investigated by circular dichroism. Panel A shows the CD spectra of calf thymus DNA (black line, at 10 μmol L^–1^ in base pairs) and **GRPA** complex (gray line, at 20 μmol L^–1^) and mixtures of DNA and metal complex at a concentration of 2, 5, 7, 10, 15, 20, and 25 μmol L^–1^. Panel B shows CD spectra of calf thymus DNA (black line) and **GRBA** metal complex (gray line) and mixtures of DNA and this metal complex as specified above.

Circular dichroism (CD) was used to provide more information on the interaction of the metal complexes with DNA. This technique allows us to analyze changes in the secondary structure of the DNA. The CD spectrum of calf thymus DNA has a positive band at 275 nm due to the stacking of the nitrogen bases and a negative band at 245 nm due to the helicity of the DNA, characteristic of DNA in the right B-form.^64,65^ By monitoring each addition of **GRPA** or **GRBA** into a cuvette containing CT-DNA, it is possible to observe their interactions with the DNA under study (Figure 8). For **GRPA**, upon increasing its concentrations, there is a decrease in the intensity of the positive band and its blue shift. These interactions indicate a weakening of the base stacking of the DNA, possibly caused by the intercalation of **GRPA** into the bases, leading to a conformational change from the B-DNA to Z-DNA form. In the negative band, there is also a decrease in intensity, indicating a loss of the right-handed helix. This profile suggests that the metal complex-DNA interaction induces a structural modification of the DNA.^66,67^

Curiously, **GRBA** did not show major changes in the CD spectrum of CT DNA, neither in the positive nor in the negative bands, but it exhibited modest changes in the intensity of CD signals. This behavior is in agreement with our previous unsuccessful attempts to measure DNA interaction with this metal compound. This behavior as seen by CD suggests a stabilization of the B-DNA form that could be due to a possible interaction with the DNA via grooves.^68^

### Photocleavage of DNA

Compounds that cause damage to DNA can be important for therapeutic purposes, however, even weakly binding compounds can still cause efficient damage, which depends on how this process occurs. Aiming to shed some light on this issue, we investigated the ability of **GRBA** and **GRPA** to cleave DNA in response to the stimulus of light. This photocleavaging study was carried out using the agarose gel electrophoresis technique, where circular pBR322 DNA (20 μmol L^–1^) along with **GRPA** or **GRBA** (from 0.5 to 10 μmol L^–1^) were irradiated with light (blue LED, λ_irrad_ = 463 nm, green LED, λ_irrad_ = 520 nm or red LED, λ_irrad_ = 693 nm) for 1 h. After this time, all samples were loaded onto an agarose gel, and an electric potential was applied for separation followed by staining and imaging.

**Figure 9:**
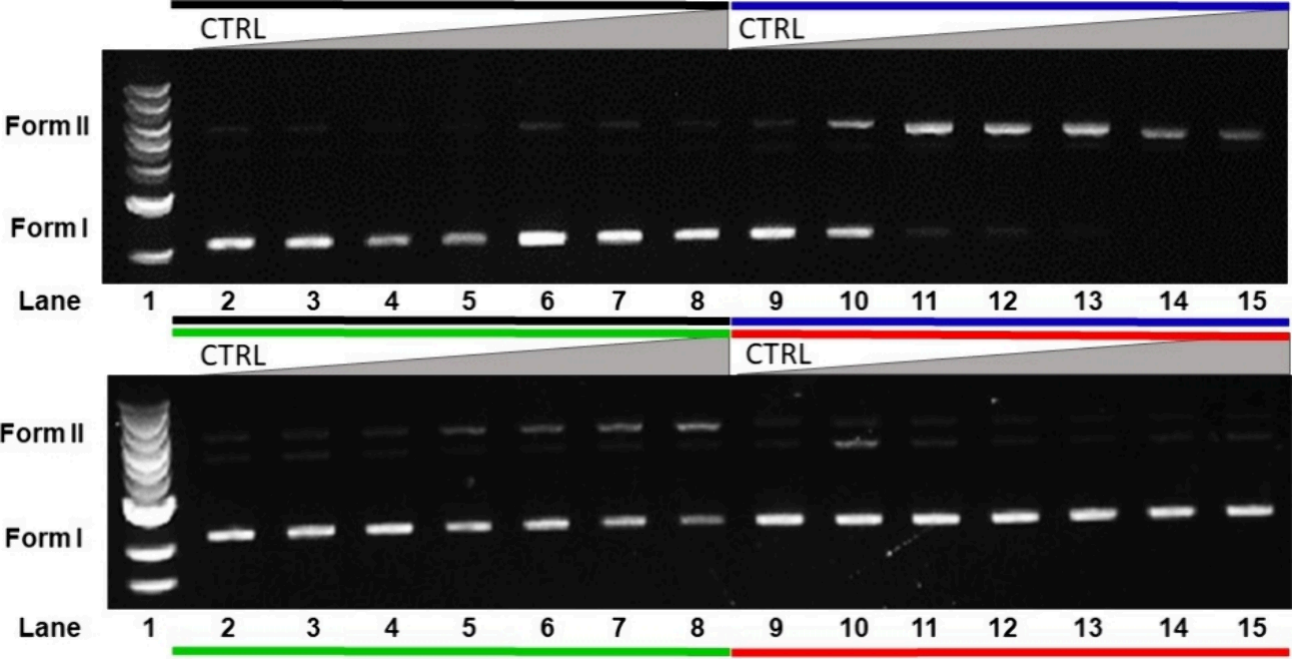
Photocleavage assay of pBR322 DNA (20 μmol L^–1^, in base pair) in the presence of **GRPA**, in the dark and after 1 h of irradiation with blue, green and red LEDs. In all experiments, lane 1 contains only linear DNA ladder (1 kb) and lane 2 only pBR322 DNA, while lanes 3–8 and 10–15 contained the following concentrations of 0.5, 1.0, 3.0, 5.0, 7.0, and 10 μmol L^–1^ of **GRPA**. Dark, blue, green and red lines indicate either the experiment was carried out in the dark or with blue, green or red-light irradiation.

First of all, our results revealed that both metal complexes incubated with DNA in the absence of light showed no evidence of DNA cleavage (Figures S23 and 9). However, upon exposure to blue and green light irradiation, both metal complexes exhibited a cleavage pattern. Notably, **GRPA** showed better efficiency in photocleaving DNA under blue light irradiation than **GRBA** (Figure S23). Even at only 0.5 μmol L^–1^ of **GRPA**, there is already damage to DNA as seen with the appearance of form II (nicked DNA), while at 1 μmol L^–1^, there is no intact DNA at all (lack of form I) being completely converted into form II (Figure 9). Regarding the experiment with **GRBA**, there is a conversion of intact DNA (form I) to nicked DNA (form II) but in a more subtle efficiency at concentrations of 0.5, 1, and 3 μmol L^–1^, whereas at 5 μmol L^–1^, it caused a complete degradation of form I (Figure S23). When subjected to green light irradiation, these metal complexes also exhibited DNA cleavage activity, but they showed moderate degradation with nicked DNA formation (form II) as observed with both metal complexes at 3 μmol L^–1^. Unfortunately, there is no DNA cleavage if red light is employed even at the maximum concentrations of the metal compounds, despite the moderate production of singlet oxygen noticed for **GRPA**.

**Figure 10:**
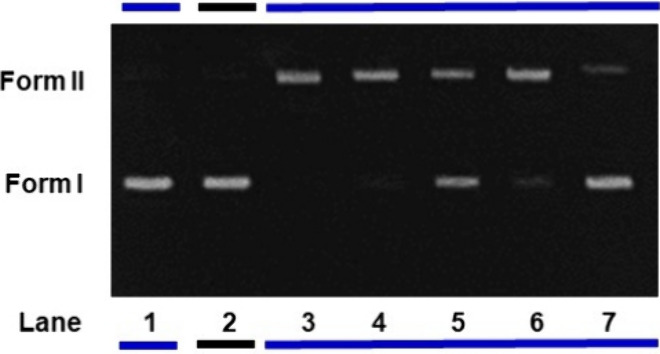
Suppression of ROS in a photocleavage assay using pBR322 DNA (20 μmol L^–1^) in the presence of **GRPA** (5 μmol L^–1^) after 1 h of blue LED irradiation, and with radical scavengers. Lane 1: pBR322 DNA only with blue light irradiation. Lane 2: DNA + complexes in the dark. Lane 3: DNA + complexes with blue light irradiation. Other lanes 4–7: pBR322 DNA + **GRPA** + suppressor: pyruvate (4), histidine (5), D-mannitol (6), and tiron (7), respectively.

Aiming to shed some light on the type of species causing DNA photocleavage, we investigated this process using standard ROS quenchers along with the metal complexes at 5 μmol L^–1^, which were irradiated with blue light for 1 h. For **GRBA** (Figure S24), a small suppression of the DNA cleavage was observed using pyruvate, histidine, and D-mannitol, which are associated with the suppression of hydrogen peroxide, singlet oxygen, and hydroxyl radical species, respectively. Some of these species can also emerge from others, such as hydrogen peroxide that can be generated from superoxide or can also yield hydroxyl radical species. Singlet oxygen production was indeed measured and its effect was expected due to its photoproduction yield of 52% in water. Interestingly, tiron caused an expressive reduction in DNA cleavage (Figure S24, lane 7), indicating that this damage is possibly mainly due to the generation of superoxide species in agreement with previous measurements. For **GRPA** (Figure 10, lane 5), there was a significant decrease in DNA cleavage in the presence of histidine (singlet oxygen suppressor). Besides that, even stronger suppression of DNA damage was noticed when tiron (superoxide anion suppressor) was used. These results indicated that **GRPA** causes DNA photocleavage mainly by the generation of ROS of the type singlet oxygen and superoxide anion. It is important to note that the photogeneration of more than one reactive species is not uncommon and occurs due to multiple photochemical deactivation routes available (Figure 3).

There is an apparent similar capacity to photodamage DNA for both metal complexes, despite their differences in the generation of ROS and binding to DNA. **GRPA** was able to photogenerate almost twice as much ^1^O_2_ but only *ca.* half of O_2_^**·**–^ and OH^**·**^ than **GRBA**. The lifetime of these radicals, level of production, relative efficacy to damage DNA along with their proximity to the target, and nature of the microenvironment (e.g., base stacking access in intercalation versus edge of bases in groove binding) should influence the overall damage. This latter issue is a relevant aspect involving the closeness to reactive targets that could influence the overall damage, particularly, considering the differences in the lifetime of ROS. How this can influence this damage when employing a moderate DNA binding (likely through intercalation) compound like **GRPA** and another one with nonmeasurable affinity to DNA (likely groove binder) as **GRBA** is an important issue. Nevertheless, these results highlighted the importance of light irradiating these metal complexes to promote DNA cleavage, as well as the influence of different reactive oxygen species on the photocleavage mechanism.

### Lipophilicity (Log *P*)

One of the fundamental parameters that influences biological processes related to drug intake, such as interactions with the target, absorption, passage through membranes, metabolism, distribution, and toxicity of compounds, is lipophilicity.^69^ This parameter is directly associated with the biological activity and biodistribution of metal complexes, implicated in its cellular uptake as well.

This lipophilicity parameter can be quantified as the logarithm of the partition coefficient of a molecule between an organic (octanol) and an aqueous phase (water or buffer solution) and expressed as Log *P*. It is necessary to seek a balance between hydrophilicity and lipophilicity in order to achieve good passive permeability. Thus, Log *P* values should be moderate, ranging from 0 to 3, in order to achieve a likely good profile between solubility and permeability, expressing ideal pharmacodynamic and pharmacokinetic conditions.^70−72^

Once the metal complex has been administered, its biodistribution is essential for the treatment to be effective and exhibit lower side effects. Therefore, it is necessary to provide a metal complex with suitable cellular accessibility, seeking a longer residence time in the body, albeit not excessive. Aiming to have a hint of these properties for **GRPA** and **GRBA**, we measured Log *P* and Log *D*_7.4_, where the latter uses phosphate buffer pH 7.4 instead of water.

Interestingly, **GRPA** showed higher coefficient values (log *P* = +0.310 and Log *D*_7.4_ = 0.260) if compared to **GRBA** (log *P* = −0.018 and Log *D*_7.4_ = 0.048). This profile suggests a greater trend of these compounds moving toward the lipid phase and probably permeating biological membranes through a passive mechanism. These values are still much better than those obtained for other related metal complexes such as [Ru(bpy)_3_]Cl_2_ (Log *P* = −2.6),^73^ [Ru(phen)_3_]Cl_2_ (Log *P* = −1.5),^73^ [Ru(bpy)_2_(dppz)]Cl_2_ (Log *P* = −2.50),^74^ and [Ru(phen)_2_(dppz)]Cl_2_ (Log *P* = −1.48).^70,75^ In addition, lipophilicity can also influence biological interactions with other targets such as intercalation with DNA bases, whereby more lipophilic compounds can have a greater affinity for DNA due to their ability to penetrate hydrophobic regions of this macromolecule.^76^ As shown further (see *Biological activities–cytotoxicity assays*), **GRPA** exhibited higher cytotoxicity than **GRBA** against mammalian cells in experiments carried out in the dark, which might indicate a better uptake by those cells. Indeed, **GRPA** showed a higher Log *P* and Log *D* in comparison not only to **GRBA** but also to many other related compounds as listed before. Nevertheless, we must always use cautiously these data because it may be only one aspect contributing to the biological activity.

### Biological Activities

#### Antimicrobial Activity

First of all, we looked at the antibiotic activity of these metal compounds aiming to access their potential use in photodynamic antimicrobial chemotherapy (PACT). Here, we explored the potential antimicrobial activity of the **GRPA** and **GRBA** complexes investigating their minimum inhibitory concentration (MIC) and minimum bactericidal concentration (MBC) against the bacteria: *S. aureus*, *S. epidermidis*, *P. aeruginosa*, and *E. coli*. This study was carried out with and without previous blue light irradiation for 1 h. Our results showed that both metal complexes did not have any measurable antibacterial activity against the Gram-negative strains used (*P. aeruginosa* and *E. coli*), neither in the dark nor upon light irradiation. This might be attributed to the presence of a more complex cell wall structure, reducing the capacity of these metal complexes to reach bacterial cytosol.

On the other hand, we observed impressive bacteriostatic and bactericidal activities with all Gram-positive bacterial strains (*S. aureus* and *S. epidermidis*), but only if light irradiated. This behavior is highly desirable in phototherapy, where the compound is expected to exhibit biological activity only upon light stimulation, allowing a very precise area of the body being treated (e.g., mouth or skin). MIC and MBC values for **GRPA** and **GRBA** ranged from 1.9 to 3.9 μg mL^–1^, in which **GRBA** showed improved results for the *S. aureus* ATCC 25923 strain (Table 3). We observed a remarkable increase in antimicrobial activity in the presence of light irradiation of over 131-fold for the *S. epidermidis* ATCC 12228 strain for both metal complexes and a similar profile for the *S. aureus* ATCC 700698 strain with **GRPA**. It is important to remark that *S. aureus* ATCC 700698 is a bacterium strain resistant to methicillin isolated from the sputum of a lung cancer patient with pneumonia and *S. epidermidis* ATCC 35984 was isolated from a case of catheter sepsis. Currently, we are facing a global crisis with bacterial infections mainly due to the emergence of multiple drug-resistant microbes. The indiscriminate use of antibiotics is among the driving forces for this phenomenon, which could be highly minimized if those antibiotics could only function under well-controlled stimuli, for example, using light. By this strategy, light-activated antibiotics if unproperly disposed of or excreted from the body into the environment would not be fully capable of inducing resistance once it requires proper light irradiation. Of course, we do not suggest all antibiotics would be a PACT, but those that are based on this strategy could be also beneficial to minimize environmental pollution, an increasing issue for current antibiotics. A possible treatment using our compound and light could be employed to reduce contamination of medical devices or equipment (e.g., catheter), commonly subject to nosocomial bacteria contamination such as *S. epidermidis* ATCC 35984. These results highlight the significant photoselectivity of these metal complexes against Gram-positive bacteria, reinforcing their potential as antimicrobial agents.

**Table 3:** Minimum Inhibitory Concentration (MIC) and Minimum Bactericidal Concentration (MBC) Values of the Ruthenium Complexes and Antibiotics (Ampicillin and Tetracycline) against Gram-Positive and Gram-Negative Bacteria with Blue Light Irradiation^a^

bacteria	ruthenium complexes μg mL^–1^ (μmol L^–1^)				antibiotics μg mL^–1^ (μmol L^–1^)			
	GRPA		GRBA		tetracycline		ampicillin	
	MIC	MBC	MIC	MBC	MIC	MBC	MIC	MBC
*S. aureus* ATCC 25923	3.9 (3.41)	3.9 (3.41)	1.9 (1.74)	1.9 (1.74)	0.39^S^ (0.87)	3.12 (6.96)	0.39^S^ (1.11)	0.78 (2.22)
*S. aureus* ATCC 700698	1.9 (1.66)	1.9 (1.66)	1.9 (1.74)	1.9 (1.74)	100^R^ (225)	100 (225)	50^R^ (143.1)	50 (143.1)
*S. epidermidis* ATCC 12228	1.9 (1.66)	3.9 (3.41)	1.9 (1.74)	1.9 (1.74)	0.39^S^ (0.87)	3.12 (6.96)	1.56^S^ (4.46)	1.56 (4.46)
*S. epidermidis* ATCC 35984	3.9 (3.41)	3.9 (3.41)	3.9 (3.57)	3.9 (3.57)	0.19^S^ (0.43)	3.12 (6.96)	1000^R^ (2862)	1000 (2862)
*E. coli* ATCC 11303	N.D.	N.D.	N.D.	N.D.	0.78^S^ (1.74)	3.12 (6.96)	0.78^S^ (2.22)	1.56 (4.46)
*P. aeruginosa* ATCC 27853	N.D.	N.D.	N.D.	N.D.	62.54^R^ (140.6)	125 (281.2)	250^R^ (715.5)	N.D.

a There is no measurable MIC or MBC in the dark even using the maximum concentration of 1000 μg mL^–1^ (^S^): Bacterium considered sensitive to ampicillin or tetracycline; (^R^): Bacterium considered resistant to ampicillin or tetracycline.^77^ (N.D.): not detected even at the highest concentration.

Another series of investigations were carried out to evaluate if these ruthenium complexes could be beneficial if used in combination with common antibiotics. The selected Gram-positive bacterial strains showed susceptibility indices resistant to the antibiotics applied (ampicillin and tetracycline), according to the recommendations and cutoff points^77^ (Table 3). *S. aureus* ATCC 700698 strain was the least susceptible to both antibiotics as measured. However, *S. epidermidis* ATCC 35984 strain exhibited a 500-fold higher MIC (1000 μg/mL) than that recommended by CLSI.^77^ Thus, this study was conducted to assess whether a combination of these antibiotics with the metal complexes could exhibit a synergistic action that might overcome current antimicrobial resistance.

**GRPA** combined with ampicillin showed an exciting synergistic effect on the strains of *S. epidermidis* ATCC 12228 and ATCC 35698 with FICI (fractional inhibitory concentration index) of 0.125, whereas *S. aureus* ATCC 25923 also showed synergism with FICI of 0.123. Only *S. aureus* ATCC 700698 in combination with ampicillin was indifferent, FICI 0.563 (Table S2). Similarly, **GRPA** combined with tetracycline showed a synergistic effect against *S. epidermidis* ATCC 12228 and *S. epidermidis* ATCC 35698 strains with FICI of 0.311 and 0.25, respectively. In this study, *S. aureus* ATCC 25923 showed a remarkable FICI of 0.123, indicating an appealing synergism with an enhancement of *ca.* 16-fold in its antibiotic action. However, this combination study showed an indifferent behavior for these compounds when used against *S. aureus* ATCC 700698 strain (FICI of 0.563) (Table S2).

Another series of studies were done using **GRBA** combined with ampicillin, where a synergistic effect was also observed with the *S. epidermidis* strain (FICI of 0.125) and *S. aureus* ATCC 25923 (FICI of 0.5). However, *S. aureus* ATCC 700698 treated in combination showed an indifferent behavior (FICI of 1.0). By looking at the results of tetracycline in combination with **GRBA**, we observed a synergistic behavior only with *S. epidermidis* ATCC 12228 (FICI of 0.313), while it was indifferent with the other strains (FICI s of 0.561 to 0.563) (Table S3). These results show a clear differential behavior for **GRPA** and **GRBA**, where **GRPA** has more promising results against these bacteria.

In summary, a great advantage of working with these metal complexes was observed in combination with some known antibiotics, which can potentiate their effects even against drug-resistant bacteria. The synergistic effect was significant when **GRPA** was combined with ampicillin and tetracycline and also **GRBA** with ampicillin.

### Cytotoxicity Assay

Further biological studies were done, where the metal complexes **GRPA** and **GRBA** were investigated to assess their potential cytotoxicity on various human carcinoma cell lines such as MDA-MB-231 (human triple-negative breast adenocarcinoma of mesenchymal phenotype), A549 (human lung alveolar epithelial basal cell adenocarcinoma), A2780 (human ovarian adenocarcinoma), and a normal healthy cell MRC-5 (human nontumorous lung). In general, both metal complexes showed cytotoxicity against all different types of cancer cells tested, with IC_50_ values ranging from micromolar to nanomolar levels, but they were highly dependent on light (Table 4).

**Table 4:** IC_50_ Values (μM) for the **GRPA** and **GRBA** Complexes in the MDA-MB-231 (Human Triple-Negative Breast Adenocarcinoma of Mesenchymal Phenotype), A2780 (Human Ovarian Adenocarcinoma), A549 (Human Lung Alveolar Epithelial Basal Cell Adenocarcinoma), and MRC-5 (Human Nontumorous Lung) Cell Lines in the Dark and upon Light Irradiation (460 nm, 10 min, 10.8 J cm^–2^) and 48 h of Incubation^a^

	cytotoxicity IC_50_ (μmol L^–1^)											
	MDA-MB-231			A2780			A549			MRC-5		
	dark	light	PI	dark	light	PI	dark	light	PI	dark	light	PI
**GRPA**	1.99 ± 0.11	0.39 ± 0.01	5.1	1.51 ± 0.03	0.23 ± 0.01	6.6	6.14 ± 0.57	1.03 ± 0.06	6.0	14.09 ± 0.98	0.34 ± 0.03	41
**GRBA**	4.48 ± 0.53	0.043 ± 0.009	104.2	3.71 ± 0.20	0.013 ± 0.005	285	16.98 ± 1.35	0.18 ± 0.02	94	>50	0.09 ± 0.01	>555

a PI = photoselectivity index (IC_50__dark/IC_50__light).

**GRPA** exhibited IC_50_ values without light irradiation of 1.51, 1.99, and 6.14 μmol L^–1^ for A2780, MDA-MB-231, and A549 cell lines, respectively. The cytotoxicity was improved upon blue light irradiation (at 460 nm for 10 min) providing IC_50_ values of 0.23, 0.39, and 1.03 μmol L^–1^ for A2780, MDA-MB-231, and A549 cell lines, respectively. This response to light meant a very modest photoselectivity index (IC_50__dark/IC_50__light) at *ca.* 6.6, 5.1, and 6.0-fold enhancement for A2780, MDA-MB-231, and A549 cell lines, respectively. Despite this, cytotoxicity against healthy MRC-5 cells in the dark was moderate (IC_50_ = 14 μmol L^–1^), meaning that only upon light irradiation, this compound can cause significant cellular damage, allowing a localized treatment as expected for phototherapy.

In the dark, **GRBA** showed IC_50_ values of 3.71, 4.48, and 16.98 μmol L^–1^ for A2780, MDA-MB-231, and A549 cell lines, respectively, which were slightly higher than those measured for **GRPA**. However, this metal complex showed a remarkable photoactivation effect with very low IC_50_ upon blue light irradiation at 0.013, 0.043, and 0.18 μmol L^–1^ for A2780, MDA-MB-231, and A549 cell lines, respectively. These results showed some cytotoxicity at 13 to 43 nmol L^–1^ of concentration, which meant an enhancement in activity from 104- up to 285-fold by using blue light. For healthy MRC-5 cells, there was no measurable cytotoxicity up to 50 μmol L^–1^, meaning that this compound could be well manageable without light. This result can also be interpreted that by applying this compound we would not observe any cytotoxicity until light was irradiated, which is expected to be done in a precise region of a tumor, then causing up to a 3,800-fold activation triggering full cytotoxicity. Unfortunately, healthy cells are also going to be destroyed with light (IC_50_ = 0.090 μmol L^–1^), but this is a common issue requiring a localized light treatment to prevent healthy cells elsewhere from being affected.

Notably, **GRBA** was expressively more potent and photoactive than **GRPA**, even with lower cytotoxicity to healthy cells as well. Some reported ruthenium complexes exhibit photoactive properties, with cytotoxicity improved after light irradiation, possessing enhanced photoselectivity for therapy.^78−80^ The mechanism of action of these photoactive compounds generally involves the photoproduction of reactive oxygen species^81^ that can cause damage to the target cells.

Here, we showed that both metal complexes, **GRPA** and **GRBA**, are capable of generating reactive oxygen species, such as singlet oxygen, superoxide, and hydroxyl radicals. These species may play an important role in the cytotoxicity of these metal complexes. In the case of **GRBA**, other possible reactions in the biological matrix (e.g., stimulated by glutathione) might lead to the formation of even more cytotoxic byproducts, which could explain its greater activity compared to **GRPA**. This suggests that the biological environment may play a role in activating and potentiating the cytotoxic activity of these metal complexes. In addition, their structural differences could lead to distinct uptake and or cellular localization, leading to distinct photocytotoxicity responses. Indeed, our in vitro data would suggest **GRPA** as the expected more bioactive compound considering Log *P*, DNA binding, and high singlet oxygen yield, but **GRBA** was actually the most effective against mammalian cells.

It is important to remark on the promising results obtained for MDA-MB-231, which is an aggressive subtype of triple-negative breast cancer. **GRBA** showed an impressive IC_50_ of 43 nmol L^–1^ upon blue light irradiation. This result supports that metal complexes may have more significant therapeutic potential, especially for some cancer cells that are more challenging to treat.

Actually, there are some compounds with promising activity against this triple-negative breast cancer (MDA-MB-231), such as [Ru(dpphen)_2_(dmbpy)](PF_6_)_2_ (where dpphen is 4,7-diphenyl-1,10-phenanthroline and dmbpy is 6,6′-dimethyl-2,2′-bipyridine). This compound showed IC_50_ of 0.74 μmol L^–1^ upon 30 min of blue light irradiation with cell incubation of 72 h.^82^ In another case, a ruthenium biphosphine complex, [Ru(GA)(dppe)_2_]PF_6_ (where GA is gallic acid and dppe is 1,2-bis(diphenylphosphino)ethane) exhibited an IC_50_ of 0.84 μmol L^–1^ without any light irradiation (after 48 h of cellular incubation).^83^ Similarly, a terpyridine-based ruthenium compound, [Ru(tpy-CM)_2_]Cl_2_ (where tpy-CM is [2,2’:6′,2″-terpyridine]-4′-il)-*N*,*N*-bis(2-chloroethyl)aniline)),^84^ showed IC_50_ of 2.6 μmol L^–1^, also without light irradiation, but with a longer cellular incubation time of 72 h. By combining a bipyridine-based ruthenium compound, Δ-[Ru(bpy)_2_(HPIP)](ClO_4_)_2_ (where HPIP is (2-hydroxyphenyl)imidazo[4,5-*f*][1,10]phenanthroline), with a known anticancer drug, doxorubicin, these authors obtained an IC_50_ of 1.2 μmol L^–1^, without light and with cellular incubation time of 24 h.^85^ One of the best ruthenium compounds for the elimination of MDA-MB-231 cells was *ct*-[RuCl(CO)(dppb)(dpqQX)]PF_6_ (where dppb is (1,4-bis(diphenylphosphino)butane) and dpqQX is dipyrido[3,2-a:2′,3′-c]quinoxaline[2,3-*b*]quinoxaline), where an IC_50_ of 0.1 μmol L^–1^ was measured without any light irradiation and after a cellular incubation time of 48 h.^86^ In our case, **GRBA** has exhibited some very exciting features, where potent cytotoxicity can be achieved upon light irradiation (43 nmol L^–1^), while without light only modest cytotoxicity is noticed making it appealing for phototherapy.

The selectivity index (SI) was also calculated for these experiments, which is an important parameter for assessing the selectivity of an anticancer compound, indicating the difference in toxicity between cancerous and healthy cells. This index may not have the same importance in phototherapy, once the cytotoxicity must be low in the dark in all tissues but very high with light. Nonetheless, **GRPA** and **GRBA** showed reasonable SI values in the dark to start with (Table S4). For MDA-MB-231 cells, **GRPA** and **GRBA** showed SI of 7.1 and >11.2, respectively. Indeed, **GRBA** showed usually better SI considering that in the dark, no cytotoxicity was observed up to 50 μmol L^–1^. We should mention that our studies investigated only 4 mammalian cells and selectivity can vary widely among different types of cancer and cell lines, and further studies are needed to evaluate this profile in a broader context.

## Conclusions

Our studies highlighted a series of promising properties identified in quite simple modified ruthenium complexes, including one of them prepared over 20 years ago.^27^ The presence of a 2,2′-bipyridine modified with anthacenyl moiety seems to be fundamental to achieve higher biological and chemical photoactivities, but not enough, while a combination with auxiliary ligands can further fine-tune suitable properties (e.g., lipophilicity, DNA binding, photochemical pathways for ROS generation). Indeed, ancillary ligands (bpy or phen) were shown to modulate the energy of the excited states allowing distinct deactivation pathways to emerge, making **GRPA** a better ^1^O_2_ photogenerator while **GRBA** was of OH^**·**^/O_2_^**·**–^.

Notably, we found that **GRBA** is a remarkable potential anticancer phototherapeutic agent. MDA-MB-231 cell is a very aggressive type of breast cancer, where this compound showed an enhancement of over 100-fold in activity with blue light, achieving IC_50_ of only 43 nmol L^–1^. This was even better for ovarian cancer cells (A2780) with an IC_50_ of 13 nmol L^–1^ with blue light. In addition to that, **GRBA** also showed a 94-fold enhancement in cytotoxicity against lung cancer cells (A549). These results are even more appealing once we consider the complete lack of cytotoxicity seen in healthy lung cells in the dark (IC_50_ > 50 μmol L^–1^). A spatial and temporal use of light, even more common nowadays with the use of fiber-optic probes, can drive very selective therapies allowing such systems to achieve great success.

Antibiotics are also an important ally during cancer patient treatment. These patients are very susceptible to bacterial infection raising a major issue during the treatment and success of recovery. *S. aureus* 700698 is a methicillin-resistant bacterial strain isolated from the sputum of a lung cancer patient with pneumonia. We investigated this strain and observed a very low response to ampicillin and tetracycline (MIC of 50 μg mL^–1^ for both antibiotics). However, **GRPA** and **GRBA** were equally efficient against this bacterium upon light irradiation (MIC and MBC of 1.9 μg mL^–1^), an activity 26-fold better in eliminating bacteria than those clinically used antibiotics. The most impressive result was their photosensitivity with >130-fold enhancement with light, while there was no measurable antibacterial activity in the dark even at the highest concentration (250 μg mL^–1^). Their synergistic behavior in combination with clinical antibiotics was also very exciting, opening not only medical opportunities but also fundamental mechanistic questions to be followed up. In addition to this, our laboratory is currently exploring the potential of these metal complexes in combination with nanocarriers in order to further improve their selectivity and enhanced delivery to cancer cells, while it could optimize pharmacodynamic-pharmacokinetic properties as well.

## Supporting Information Available

^1^H, COSY, ^13^C NMR data; FTIR of GRPA and GRBA in KBr pellets; electronic absorption data (in methanol); cyclic voltammograms of GRBA and GRPA; TD-DFT contour surfaces; stability measurements monitored by electronic spectroscopy and HPLC; measurements of ROS using DPBF and SOSG in methanol; superoxide measurement using NBT; DNA binding using electronic spectroscopy; DNA binding competition studies using ethidium bromide; groove binder studies using methyl green and Hoechst; DNA photocleavage using GRBA by agarose electrophoresis; DNA photocleavage with ROS scavengers; TD-DFT studies for GRPA and GRBA; effect of GRPA and GRBA in association with antibiotics; and selectivity index (PDF)
