# Supplementary material for: Minimal Functionalization of Ruthenium Compounds with Enhanced Photoreactivity against Hard-to-Treat Cancer Cells and Resistant Bacteria
Source: Inorg Chem. 2024 Jul 23;63(31):14673–90. doi: 10.1021/acs.inorgchem.4c02235 (PMC11304396; doi:10.1021/acs.inorgchem.4c02235)
Supplement: Supplementary file 1 — ic4c02235_si_001.pdf [file ic4c02235_si_001.pdf]

Supporting Information

Minimal Functionalization of Ruthenium Compounds with Enhanced Photoreactivity against Hard-to-Treat Cancer Cells and Resistant Bacteria.

Authors: Geângela de Fátima Sousa Oliveira[1], Florencio Sousa Gouveia Jr[1], Alexandre Lopes Andrade [2], Mayron Alves de Vasconcelos [3], Edson Holanda Teixeira[2], Marcos V. Palmeira-Mello[4], Alzir A. Batista[4], Luiz Gonzaga de França Lopes[1], Idalina Maria Moreira de Carvalho[1,*] and Eduardo Henrique Silva Sousa[1,*]

1- Laboratório de Bioinorgânica, Departmento de Química Orgânica e Inorgânica, Universidade Federal do Ceará, 60440-900, Fortaleza, Brasil.

2- Laboratório Integrado de Biomoléculas, Departamento de Patologia e Medicina Legal, Universidade Federal do Ceará, 60441-750, Fortaleza, Ceará, Brasil

3- Faculdade de Educação de Itapipoca, Universidade Estadual do Ceará, Itapipoca, CE, Brazil.

4- Departamento de Química, Universidade Federal de São Carlos, PO Box 676, 13565-905, São Carlos, São Paulo, Brasil

Figure S1. ^{1}H NMR for **GRPA** (A), COSY (B) and ^{13}C (C) in $(CD_3)_2SO$.

Figure S2. ^{1}H NMR for **GRBA** in $(CD_3)_2SO$.

Figure S3. Infrared vibrational spectra of the compounds: bpy-anth (green line), $Ru(bpy)_2Cl_2$ (red line) and **GRBA** (black line) on KBr pellets.

Figure S4. Infrared vibrational spectra of the compounds: bpy-anth (green line), $Ru(phen)_2Cl_2$ (red line) and **GRPA** (black line) on KBr pellets.

Figure S5. Electronic absorption (A) and emission (B) spectra of **GRBA** in methanol (2 x 10^{-5} mol L^{-1}) at 25 ^oC (emission was measured after excitation at 446 nm).

Figure S6. Electronic absorption of mbpy-anth (black line) and Anth-NH_2 (red line) in methanol at 25 ^oC.

Figure S7. Cyclic voltammogram of **GRPA** in acetonitrile (0.1 mol L^{-1} PTBA, 100 mV s^{-1}), initial anodic scan (blank in dashed blue line).

Figure S8. Cyclic voltammogram of **GRBA** in acetonitrile (0.1 mol L^{-1} PTBA, 100 mV s^{-1}), initial anodic scan (blank in dashed blue line).

Figure S9. Contour surfaces of the molecular orbitals of the **GRBA** complex calculated by TD-DFT.

Figure S10. Contour surfaces of the molecular orbitals of the **GRPA** complex calculated by TD-DFT.

Figure S11. Stability study of the complexes monitored by electronic absorption spectroscopy. Panels A and C show the spectra of **GRPA** monitored for 48h in the dark and for 270 min irradiated with blue light, respectively. Panels B and D show the spectra of **GRBA** monitored for 48h in the dark and monitored for 270 min irradiated with blue light, respectively.

Figure S12. Stability study of the **GRBA** complex was monitored by HPLC for 48 hours, with injections of 10 μL using a mixture of 15% methanol in water containing 0.1% of NaTFA (sodium trifluoroacetate) at pH 3.5 as mobile phase, with a flow rate of 1 mL min^{-1}.

Figure S13. Stability study of the **GRBA** complex monitored by HPLC for 1 hour irradiated with blue light (A), precursor complex $[Ru(bpy)_2Cl_2]$ (B), and precursor complex $[Ru(bpy)_2(mbpy\text{-}COOH)]^{2+}$ (C) a mixture of 15% methanol in water containing 0.1% of NaTFA (sodium trifluoroacetate) at pH 3.5 as mobile phase, with a flow rate of 1 mL min^{-1}.

Figure S14. Stability study of the **GRPA** complex monitored by HPLC for 48 hours, with injections of 20 μL in a mixture of 15% methanol in water containing 0.1% of NaTFA (sodium trifluoroacetate) at pH 3.5 as mobile phase, with a flow rate of 1 mL min^{-1}.

Figure S15. Stability study of the **GRPA** complex monitored by HPLC for 1 hour irradiated with blue light (A), precursor complex [Ru(phen)$_2$Cl$_2$] (B), and precursor complex [Ru(phen)$_2$(mbpy-COOH)]$^{2+}$ (C) in a mixture of 15% methanol in water containing 0.1% of NaTFA (sodium trifluoroacetate) at pH 3.5 as mobile phase, with a flow rate of 1 mL min^{-1}.

Figure S16. Singlet oxygen photoproduction measured using DPBF as a probe. Panels A, B and C show emission spectra of DPBF with **GRPA** upon irradiation with blue, green and red light, respectively. Panels D, E and F show time-dependence curves for quantum yield measurements of samples irradiated with blue, green and red light, respectively. The red circles are for DPBF alone, black squares for **GRPA**, green diamond for **GRBA** and blue tringles for standards (for blue light, [Ru(bpy)$_3$]$^{2+}$ (Φ_Δ = 0.87) was used, while for green and red light, rose bengal (Φ_Δ = 0.76) and methylene blue (Φ_Δ = 0.50), respectively, in methanol, at 25 ^{o}C.

Figure S17. Measurement of the production of singlet oxygen upon blue light irradiation using SOSG probe (1 μmol L^{-1}) in methanol (λ_{exc} at 490 nm). Panel A shows a linear change of the fluorescence during blue light irradiation in methanol for SOSG (alone, black circle), **GRPA** (green inverted triangle), **GRBA** (red triangle) and [Ru(bpy)$_3$]$^{2+}$ (blue square) . Panel B, C and D show emission spectra for SOSG with **GRPA** (10 μmol L^{-1}), **GRBA** (10 μmol L^{-1}) and [Ru(bpy)$_3$]$^{2+}$ (10 μmol L^{-1}) during blue light irradiation.

Figure S18. Superoxide production study employing **NBT** (50 μmol L^{-1}) with **GRBA** (5μmol L^{-1}) (A) or **GRPA** (5μmol L^{-1}) (B), NBT with GSH only (1.5 mmol L^{-1}) (C) and and the graph of Abs590 x time of the GRPA and GRBA complexes (D), all with blue light irradiation.

Figure S19. DNA binding measurements. Panels show the titration of **GRBA** with calf thymus DNA monitored by UV-vis absorption electronic spectra (A) and luminescence with excitation at 450 nm (B), in 0.1 mmol L^{-1} Tris-HCl pH 7.4) at 25 ^{o}C.

Figure S20. Competition assay of ethidium bromide (1.5 μmol L^{-1}) with DNA upon titration with **GRPA**.

Figure S21 Emission spectra of Methyl Green (red line) in the presence of CT-DNA (black line) at different metal complex concentrations (0-20 μmol L^{-1}), **GRPA** (A) and **GRBA** (B). [Methyl Green] = 5 μmol L^{-1} and [CT DNA] = 10 μmol L^{-1}, λ_{exc}= 340 nm.

Figure S22. Emission spectra of Hoechst (blue line) in the presence of CT-DNA (black line) at different metal complex concentrations (0-28 μmol L^{-1}), **GRPA** (A) and **GRB**A (B). [Hoechst] = 5 μmol L^{-1} and [CT DNA] = 10 μmol L^{-1}, λ_{exc}= 340 nm.

Figure S23. Photocleavage of 20 μM (in base pair) pBR322 DNA in the presence of **GRBA** in the dark and after 1 h of irradiation with blue, green and red LEDs. In all experiments, lane 1

contains only linear DNA ladder and lane 2 has only pBR322 DNA, while lanes 3−8 and 10−15 contained the following concentrations of 0.5, 1.0, 3.0, 5.0, 7.0 and 10 μmol L^{-1} of **GRBA**. Dark, blue, green and red lines indicate either the experiment was carried out in the dark or with blue, green or red-light irradiation.

Figure S24. Photocleavage of pBR322 DNA (20 μmol L^{-1}) in the presence of **GRBA** (5 μmol L^{-1}) after 1 h of blue LED irradiation in the presence of radical scavengers. Lane 1: pBR322 DNA only with blue irradiation. Lane 2: DNA + complexes in the dark. Lane 3: DNA + complexes with blue light irradiation. Lanes 4 – 7: pBR322 DNA + **GRBA** + suppressors pyruvate (4), histidine (5), D-mannitol (6) and tiron (7), respectively.

Table S1. TD-DFT Wavelengths (oscillator strengths) and characters of the spin-allowed electronic transitions assigned of selected transitions for **GRPA** and **GRBA** in methanol media.

Table S2 – Effect of **GRPA** associated with ampicillin (AMP) and tetracycline (TETRA) antibiotics against *S. aureus* and *S. epidermidis*.

Table S3 - Effect of the **GRBA** complex associated with AMP and TETRA antibiotics on *S. aureus* and *S. epidermidis*.

Table S4. Selectivity index (SI) of the metal compounds for different tumor cell lines through the ratio SI = IC_{50}(MRC-5)/IC_{50}(tumor cell).

A

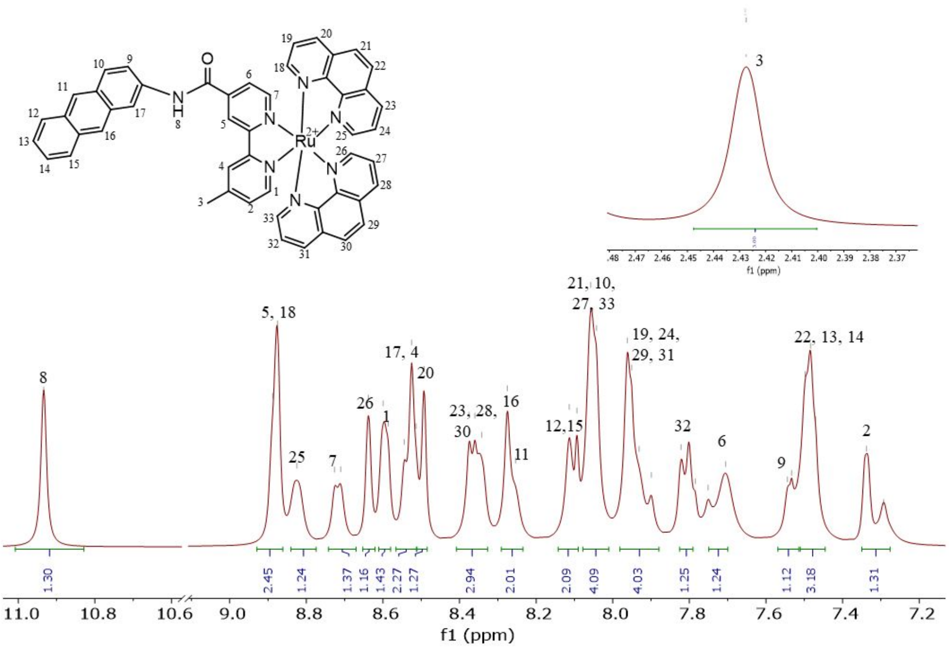

B

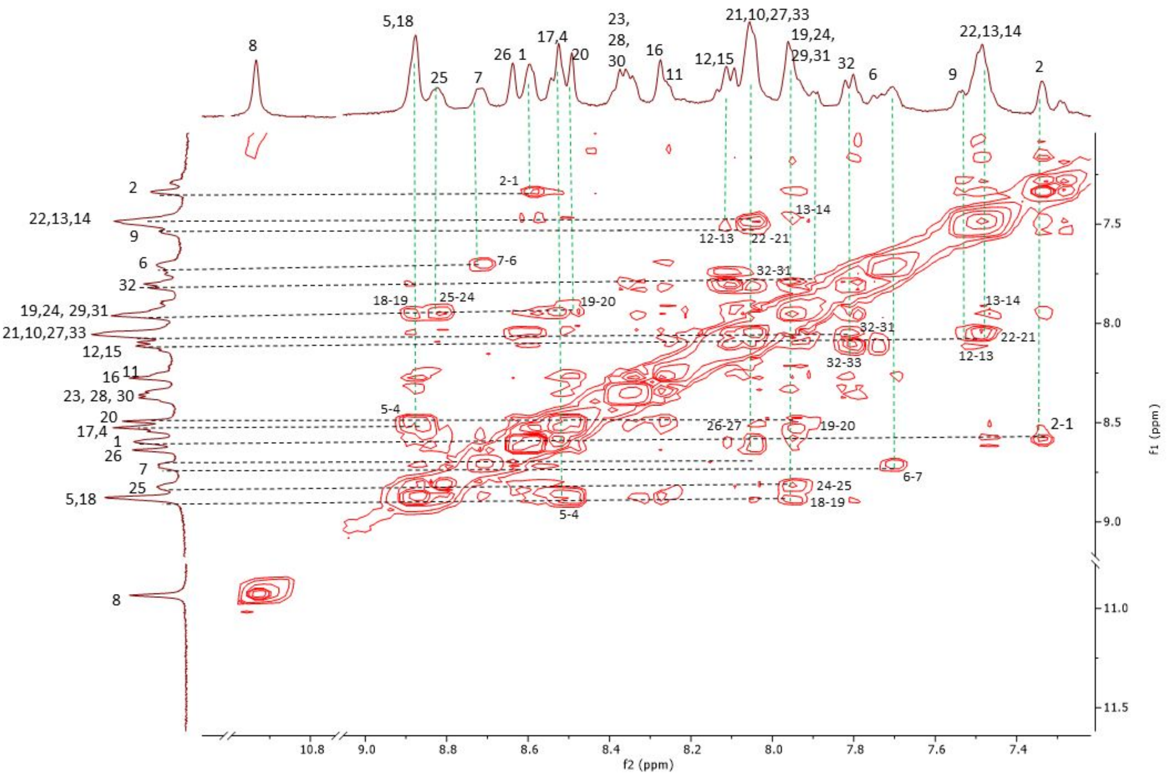

C

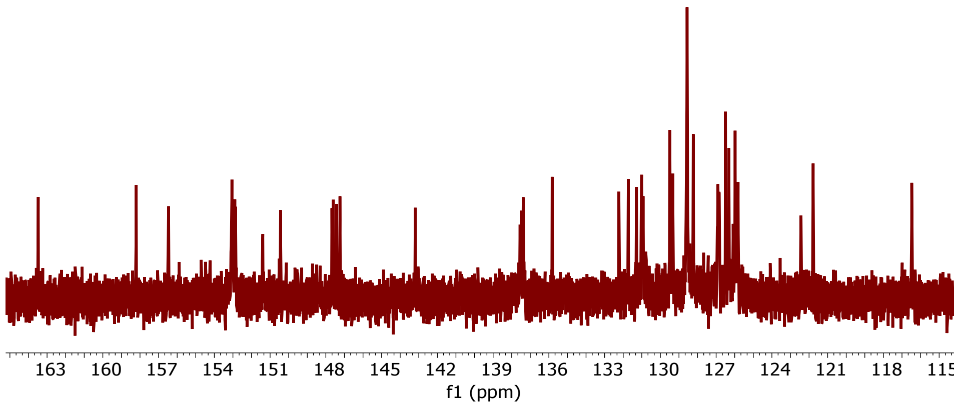

Figure S1. ^{1}H NMR for **GRPA** (A), COSY (B) and ^{13}C (C) in $(CD_3)_2SO$.

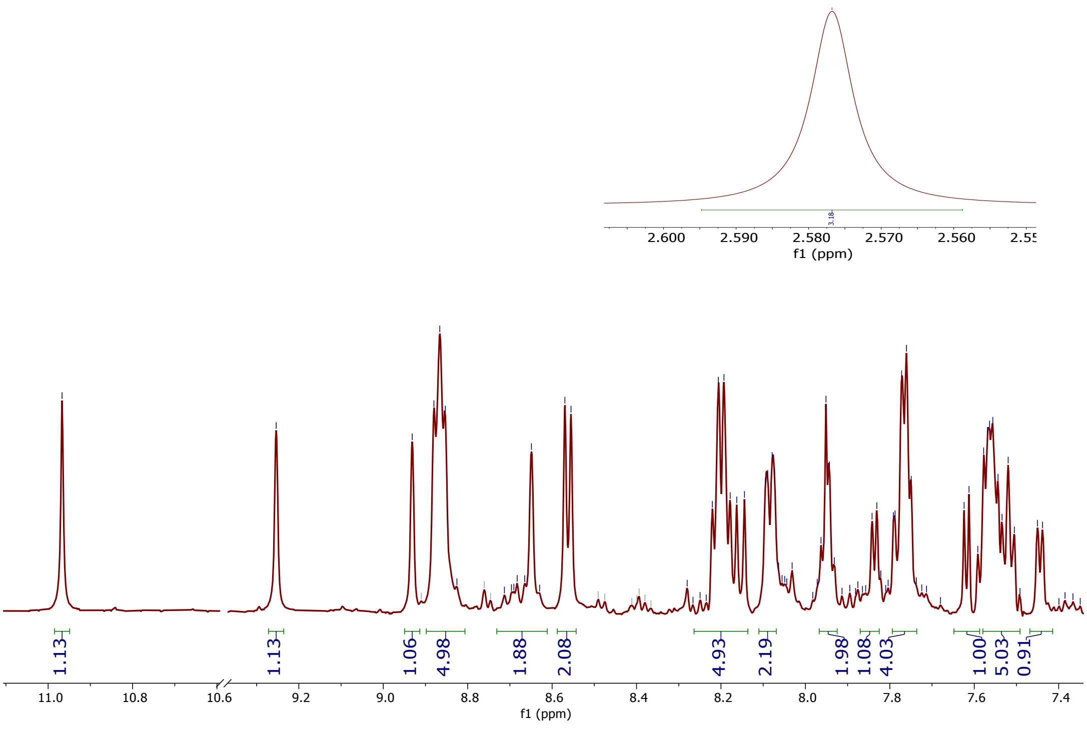

Figure S2. ^{1}H NMR for **GRBA** in $(CD_3)_2SO$.

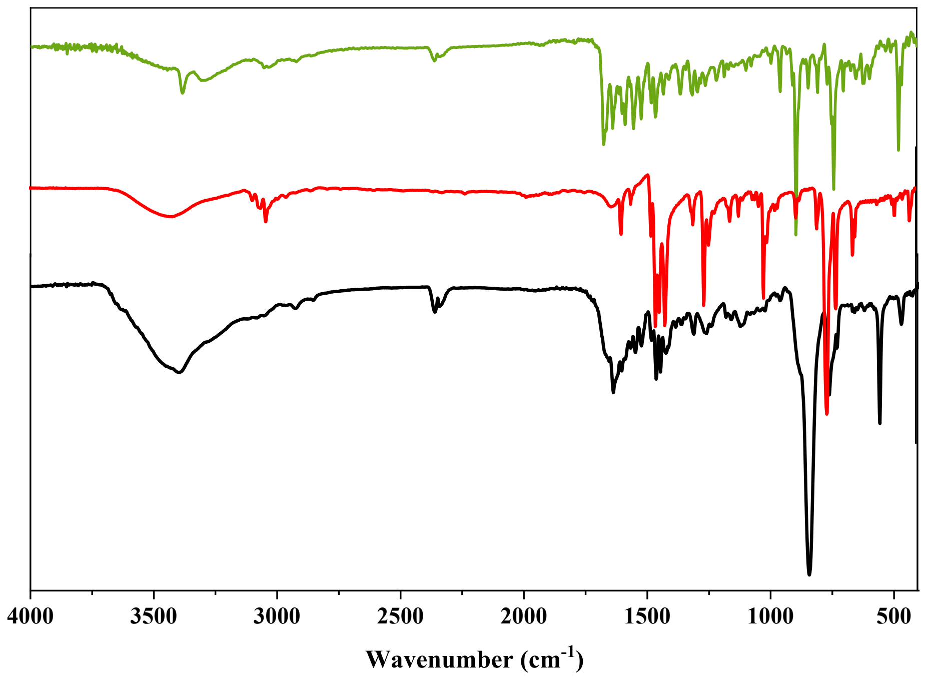

Figure S3. Infrared vibrational spectra of the compounds: bpy-anth (green line), Ru(bpy)$_2$Cl$_2$ (red line) and **GRBA** (black line) on KBr pellets.

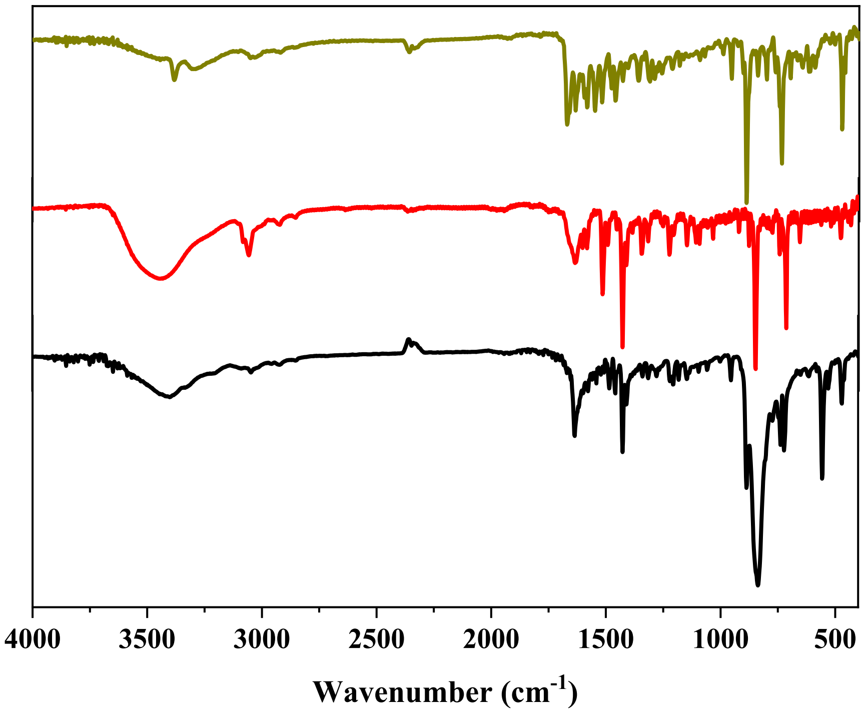

Figure S4. Infrared vibrational spectra of the compounds: bpy-anth (green line), Ru(phen)$_2$Cl$_2$ (red line) and **GRPA** (black line) on KBr pellets.

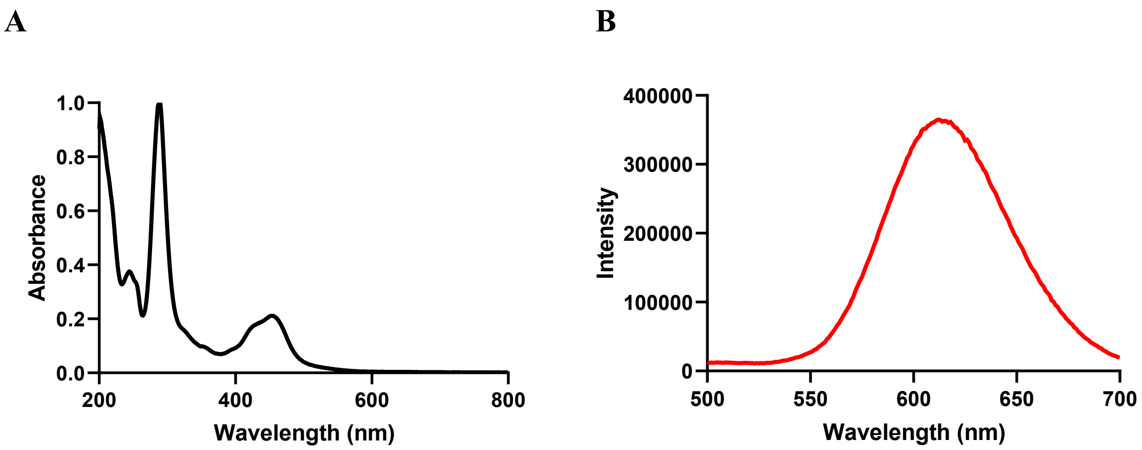

Figure S5. Electronic absorption (A) and emission (B) spectra of **GRBA** in methanol (2 x 10^{-5} mol L^{-1}) at 25 ^{o}C (emission was measured after excitation at 446 nm).

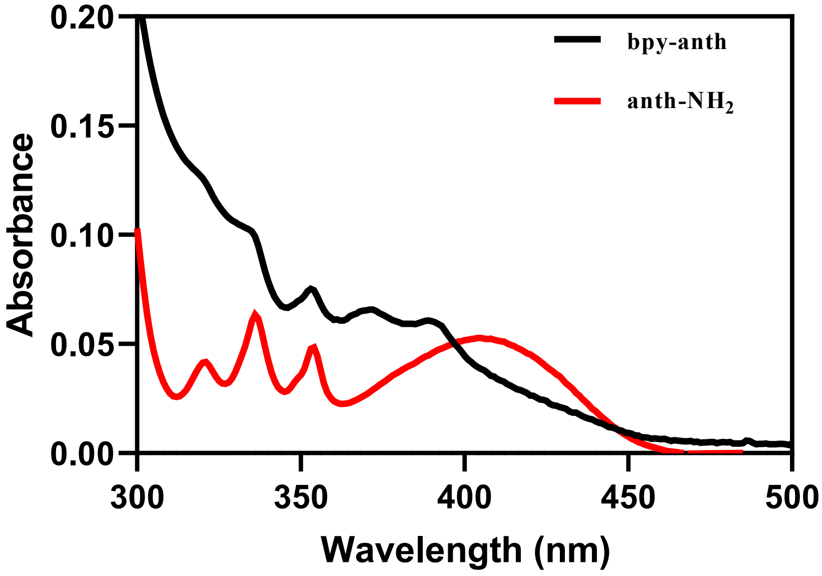

Figure S6. Electronic absorption of mbpy-anth (black line) and Anth-NH_2 (red line) in methanol at 25 ^{o}C.

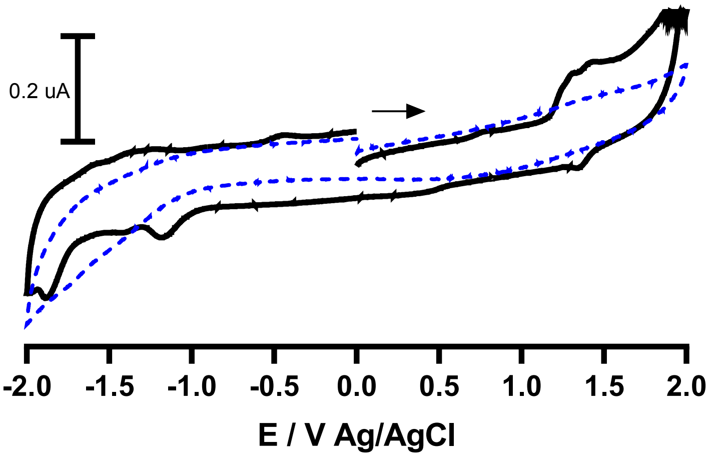

Figure S7. Cyclic voltammogram of **GRPA** in acetonitrile (0.1 M PTBA, 100 mV s^{-1}), initial anodic scan (blank in dashed blue line) (value of ferrocene redox pair of 0.410 V).

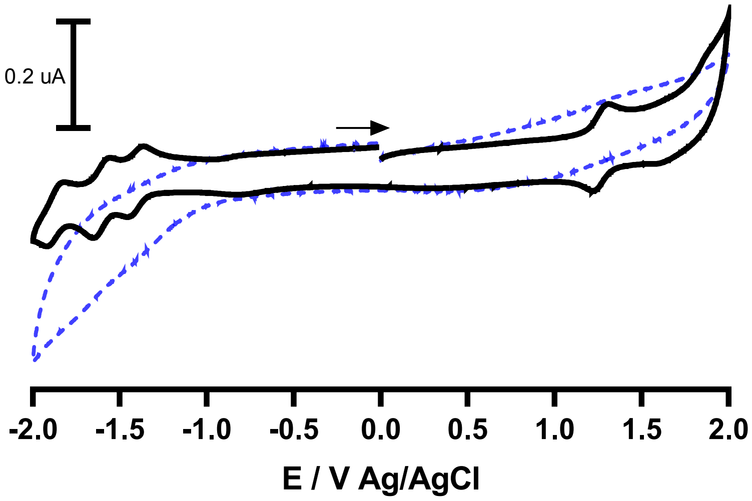

Figure S8. Cyclic voltammogram of **GRBA** in acetonitrile (0.1 M PTBA, 100 mV s^{-1}), initial anodic scan (blank in dashed blue line) (value of ferrocene redox pair of 0.410 V).

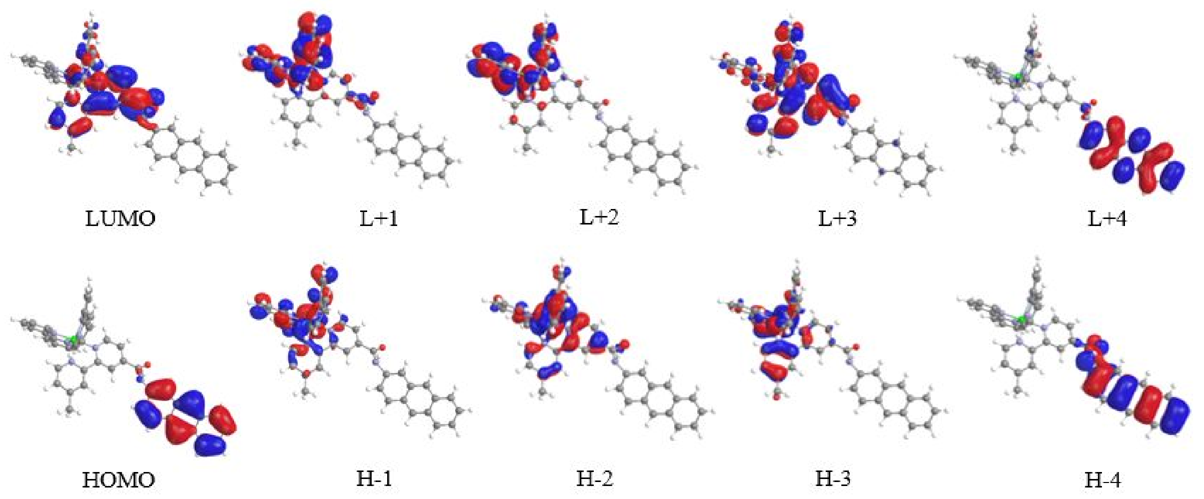

Figure S9. Contour surfaces of the molecular orbitals of the **GRBA** complex calculated by TD-DFT.

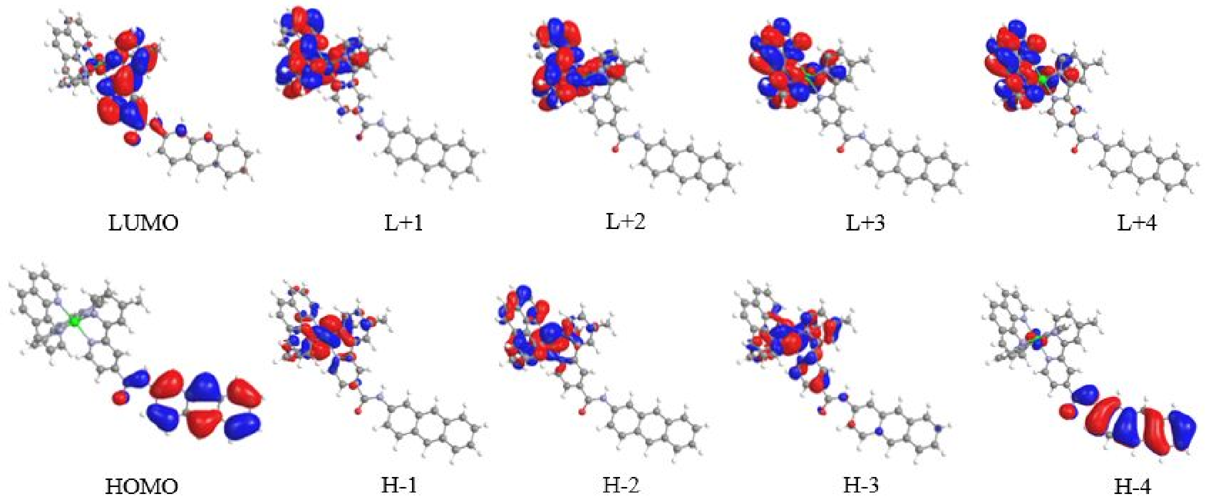

Figure S10. Contour surfaces of the molecular orbitals of the **GRPA** complex calculated by TD-DFT.

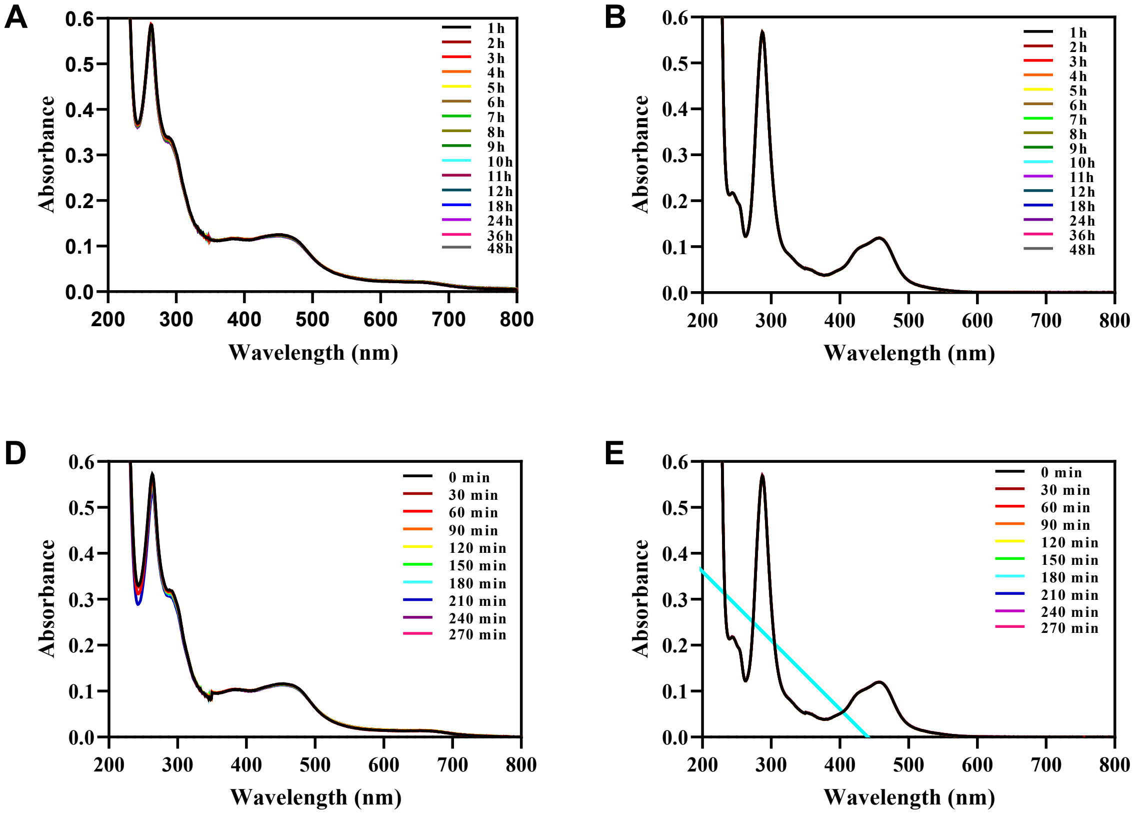

Figure S11. Stability study of the complexes monitored by electronic absorption spectroscopy. Panels A and C show the spectra of **GRPA** monitored for 48h in the dark and for 270 min irradiated with blue light, respectively. Panels B and D show the spectra of **GRBA** monitored for 48h in the dark and monitored for 270 min irradiated with blue light, respectively.

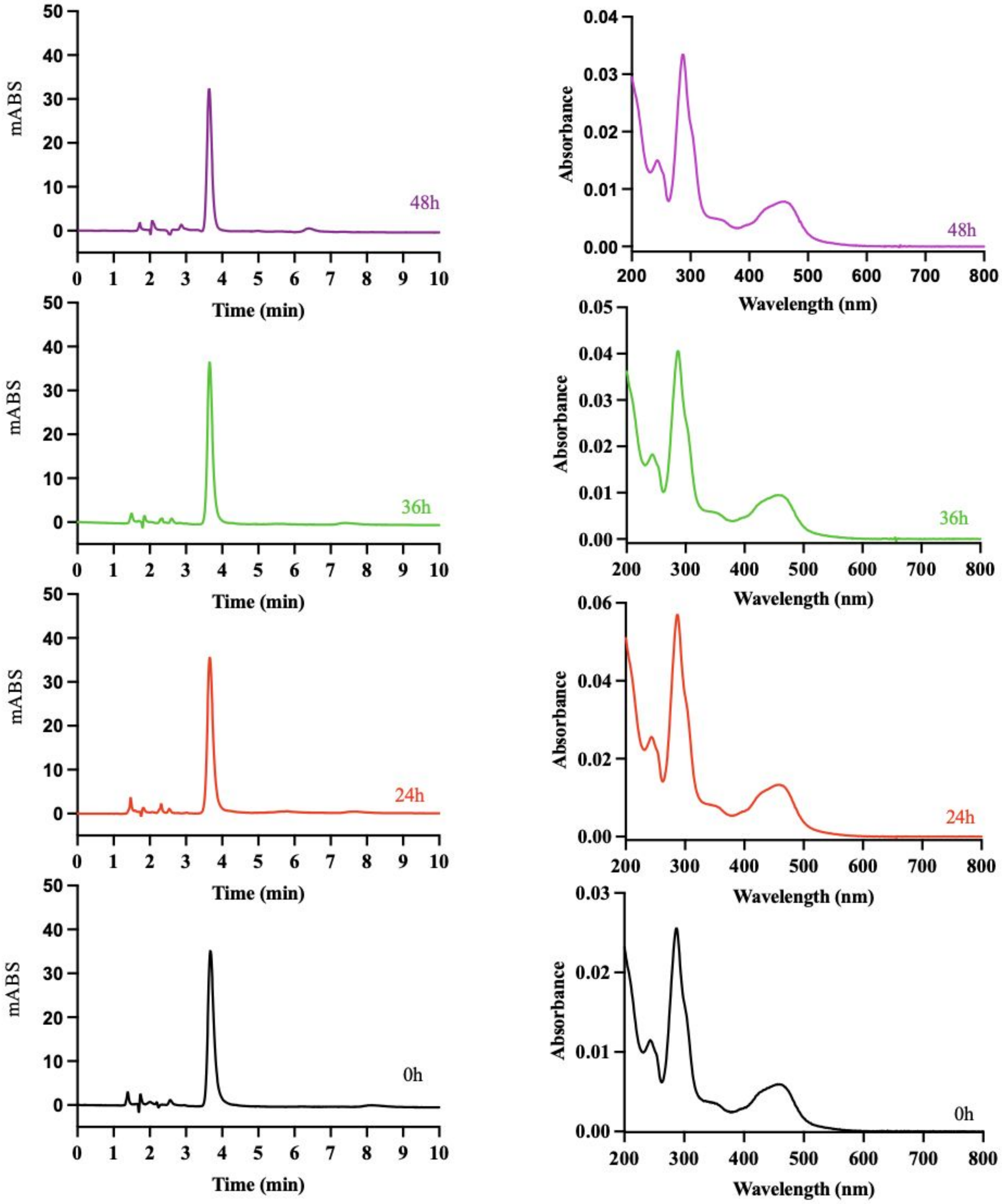

Figure S12. Stability study of the **GRBA** complex monitored by HPLC for 48 hours, with injections of 10 uL in a 15% acetonitrile and 85% 0.1% NaTFA running phase.

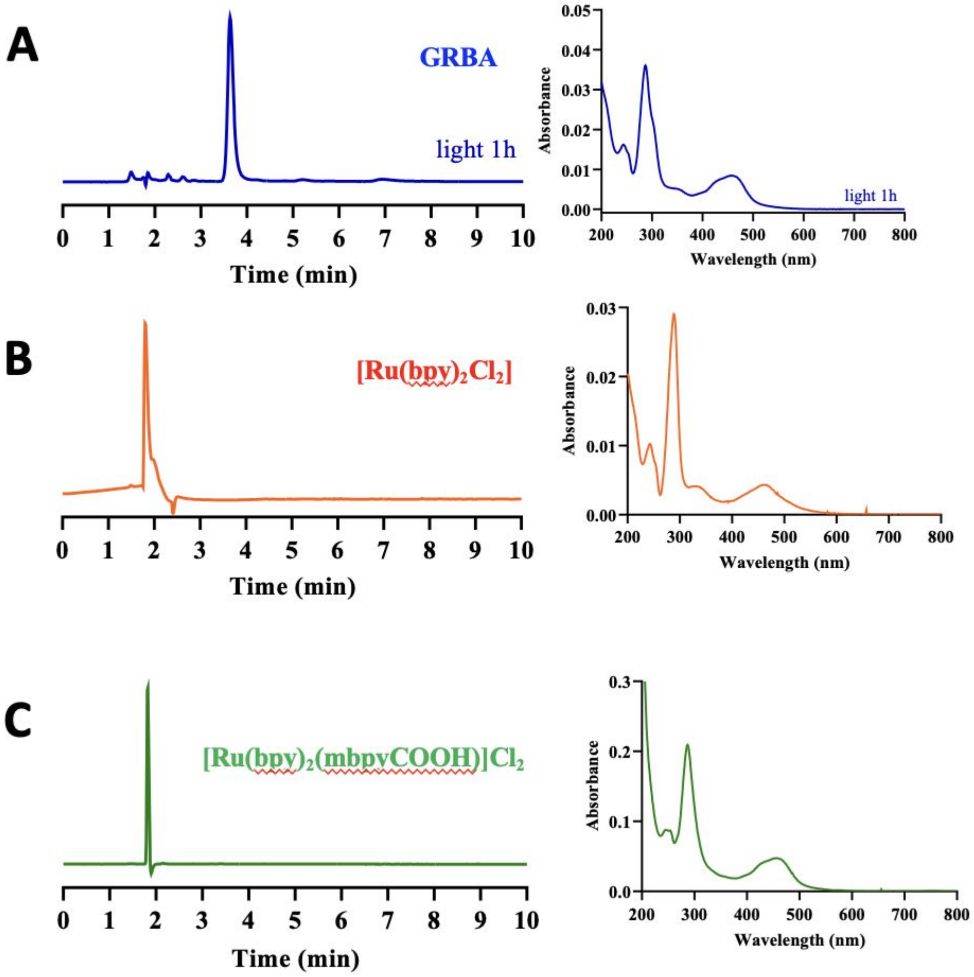

Figure S13. Stability study of the **GRBA** complex monitored by HPLC for 1 hour irradiated with blue light (A), precursor complex [Ru(bpy)$_2$Cl$_2$] (B), and precursor complex [Ru(bpy)$_2$(mbpy-COOH)]$^{2+}$ (C) in a 15% acetonitrile and 85% 0.1% NaTFA running phase.

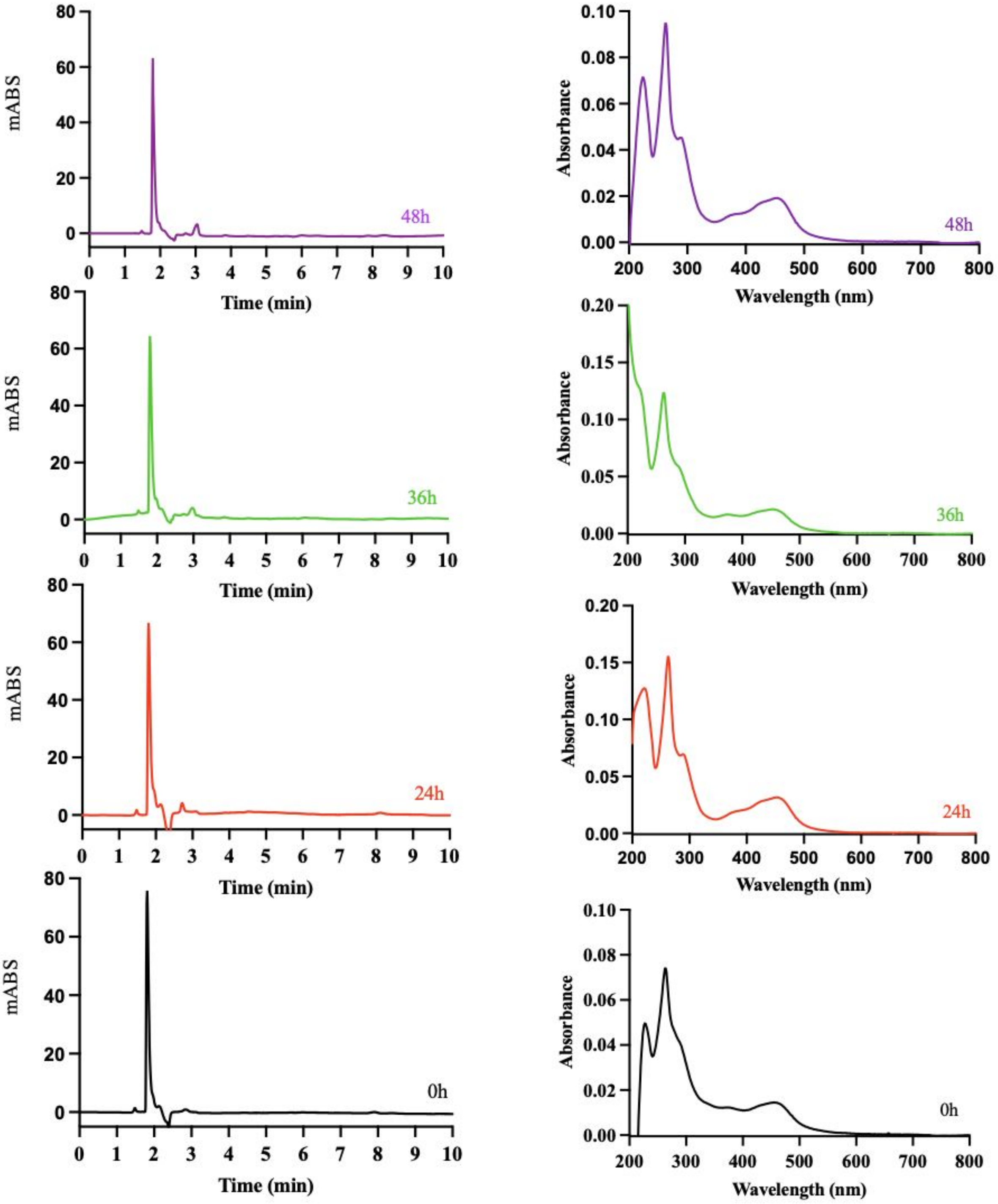

Figure S14. Stability study of the **GRPA** complex monitored by HPLC for 48 hours, with injections of 20 uL in a 15% acetonitrile and 85% 0.1% NaTFA running phase.

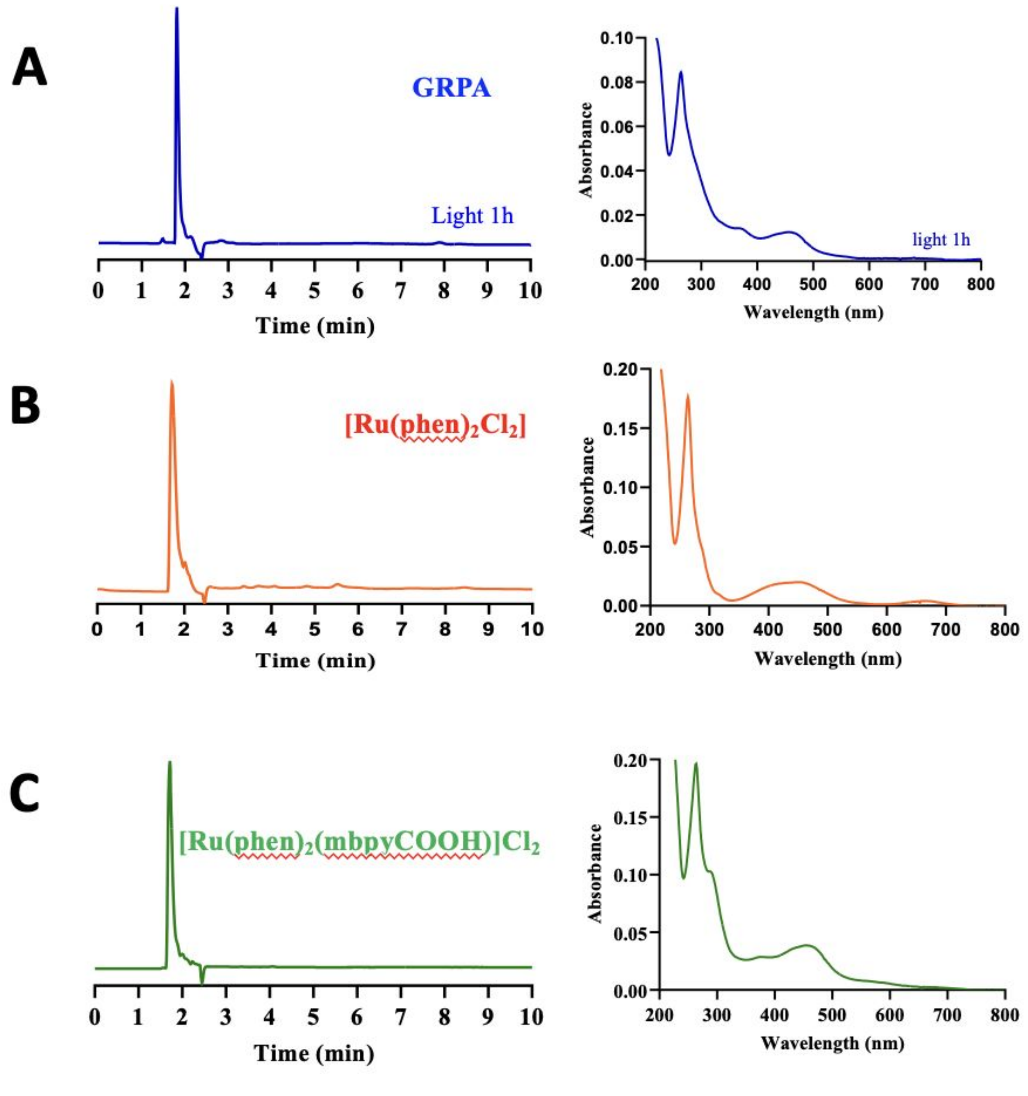

Figure S15. Stability study of the **GRPA** complex monitored by HPLC for 1 hour irradiated with blue light (A), precursor complex [Ru(phen)$_2$Cl$_2$] (B), and precursor complex [Ru(phen)$_2$(mbpy-COOH)]$^{2+}$ (C) in a 15% acetonitrile and 85% 0.1% NaTFA running phase.

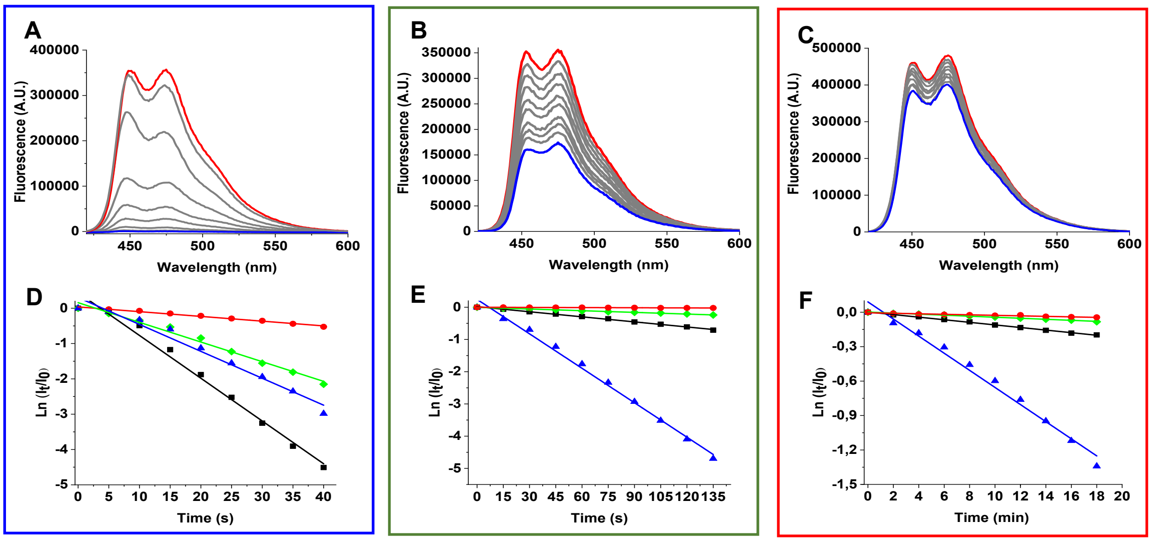

Figure S16. Singlet oxygen photoproduction measured using DPBF probe. Panels A, B and C show emission spectra of DPBF with **GRPA** upon irradiation with blue, green and red light, respectively. Panels D, E and F show time-dependence curves for quantum yield measurements of samples irradiated with blue, green and red light, respectively. The red circles are for DPBF alone, black squares for **GRPA**, green diamond for **GRBA** and blue tringles for standards (for blue light, $[Ru(bpy)_3]^{2+}$ ($\Phi_\Delta = 0.87$) was used, while for green and red light, rose bengal ($\Phi_\Delta = 0.76$) and methylene blue ($\Phi_\Delta = 0.50$), respectively, in methanol, at 25 ^oC.

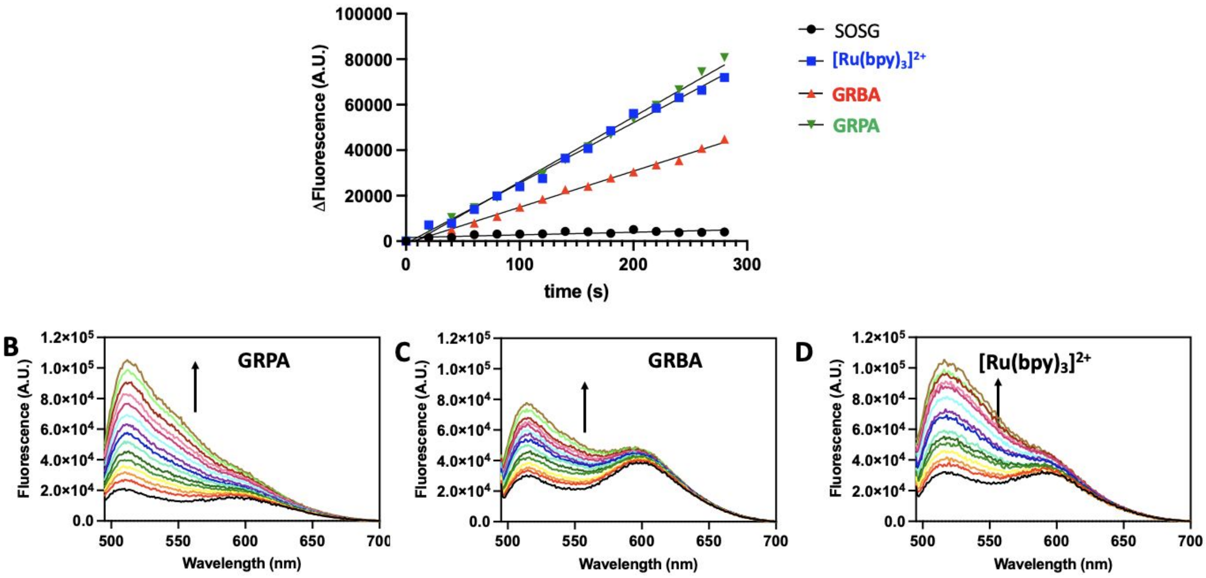

Figure S17. Measurement of the production of singlet oxygen upon blue light irradiation using SOSG probe (1 μmol L^{-1}) in methanol (λ_{exc} at 490 nm). Panel A shows a linear change of the fluorescence during blue light irradiation in methanol for SOSG (alone, black circle), **GRPA** (green inverted triangle), **GRBA** (red triangle) and $[Ru(bpy)_3]^{2+}$ (blue square) . Panel B, C and D show emission spectra for SOSG with **GRPA** (10 μmol L^{-1}), **GRBA** (10 μmol L^{-1}) and $[Ru(bpy)_3]^{2+}$ (10 μmol L^{-1}) during blue light irradiation.

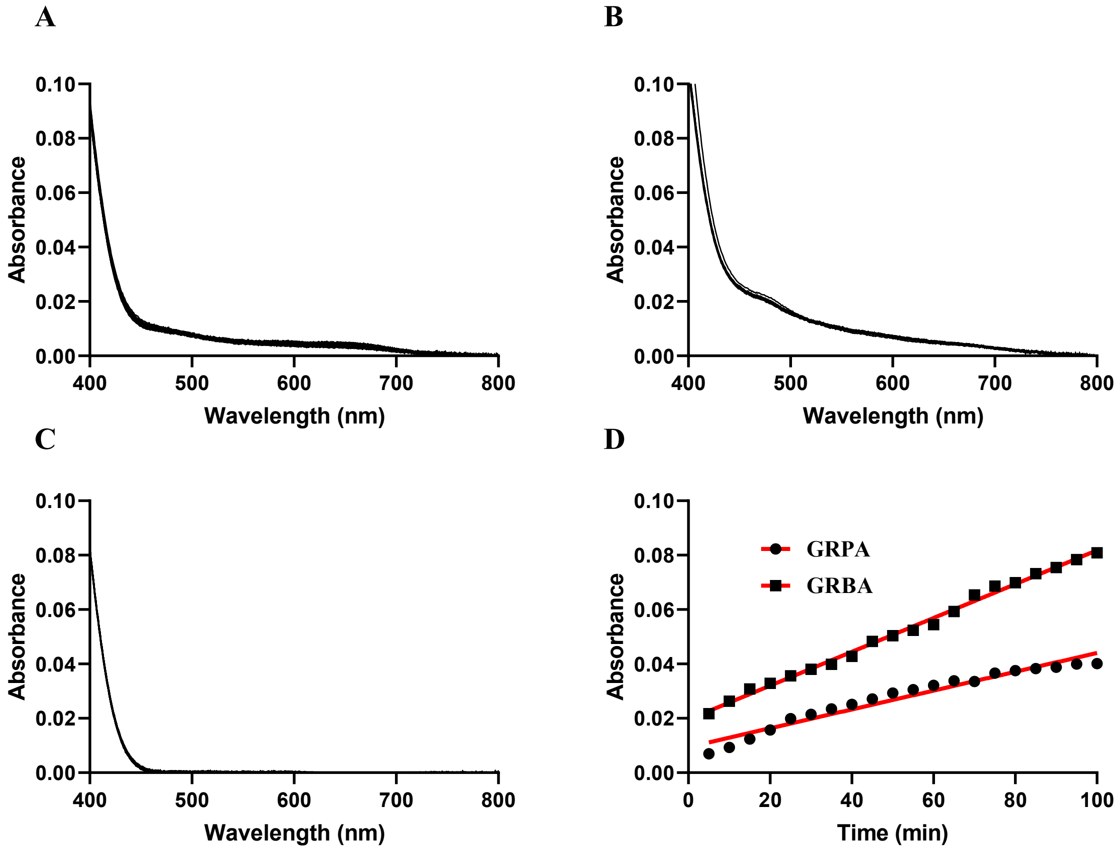

Figure S18. Superoxide production study employing **NBT** (50 µmol L^{-1}) with **GRBA** (5µmol L^{-1}) (A) or **GRPA** (5µmol L^{-1}) (B), NBT with GSH only (1.5 mmol L^{-1}) (C) and and the graph of Abs590 x time of the GRPA and GRBA complexes (D), all with blue light irradiation.

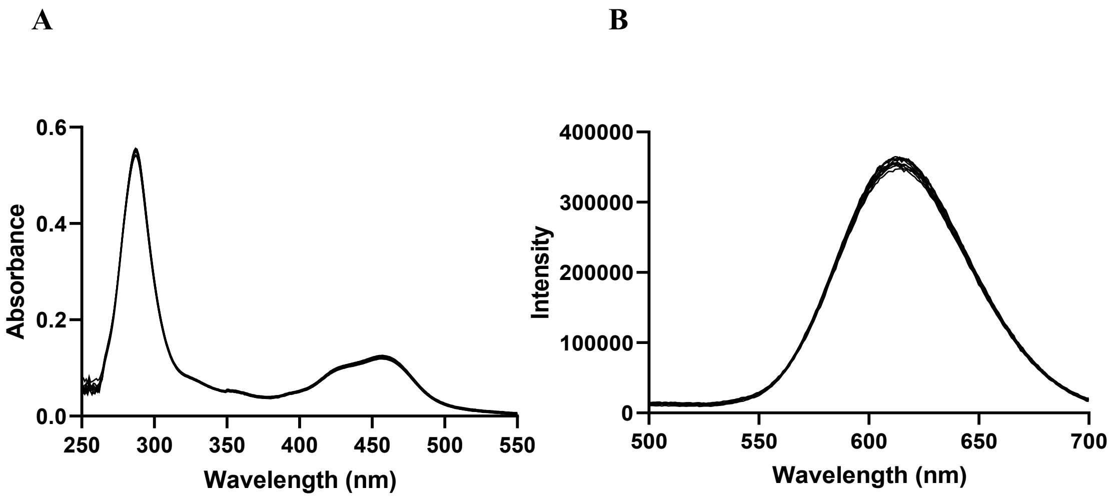

Figure S19. DNA binding measurements. Panels show the titration of **GRBA** with calf thymus DNA monitored by UV-vis absorption electronic spectra (A) and luminescence with excitation at 450 nm (B), in 0.1 mmol L^{-1} Tris-HCl pH 7.4) at 25 °C.

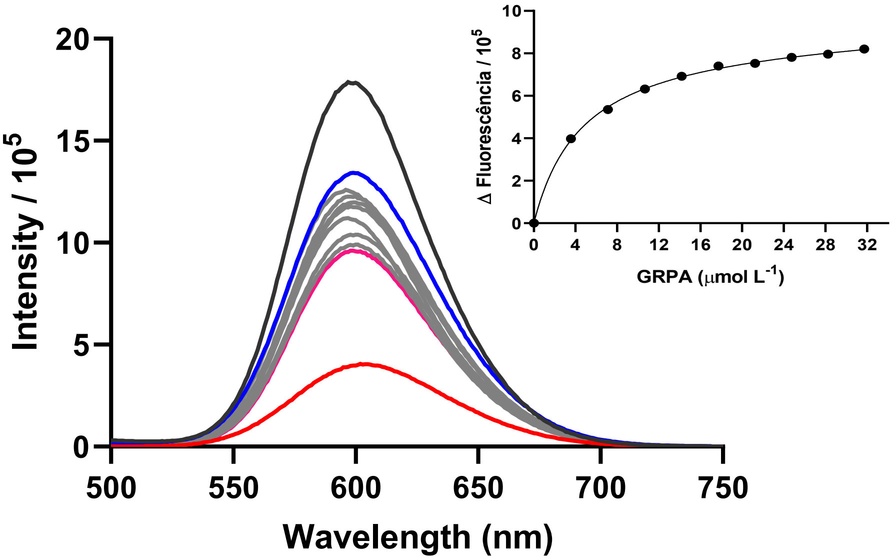

Figure S20. Competition assay of ethidium bromide (1.5 μmol L^{-1}) with DNA upon titration with **GRPA**.

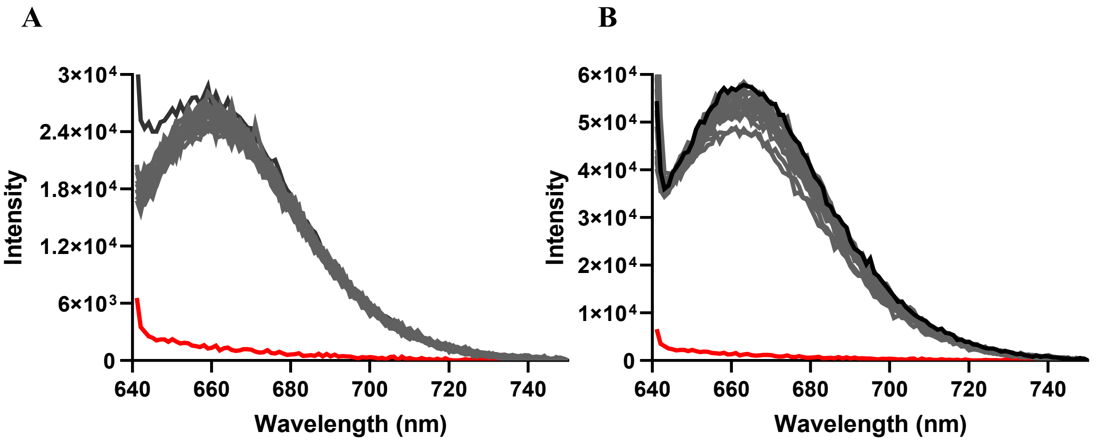

Figure S21 Emission spectrum of Methyl Green (red line) in the presence of CT-DNA (black line) at different complex concentrations (0-20 µmol L^{-1}), **GRPA** (A) and **GRBA** (B). [Methyl Green] = 5 µmol L^{-1} and [CT DNA] = 10 µmol L^{-1}, λ_{exc}= 340 nm.

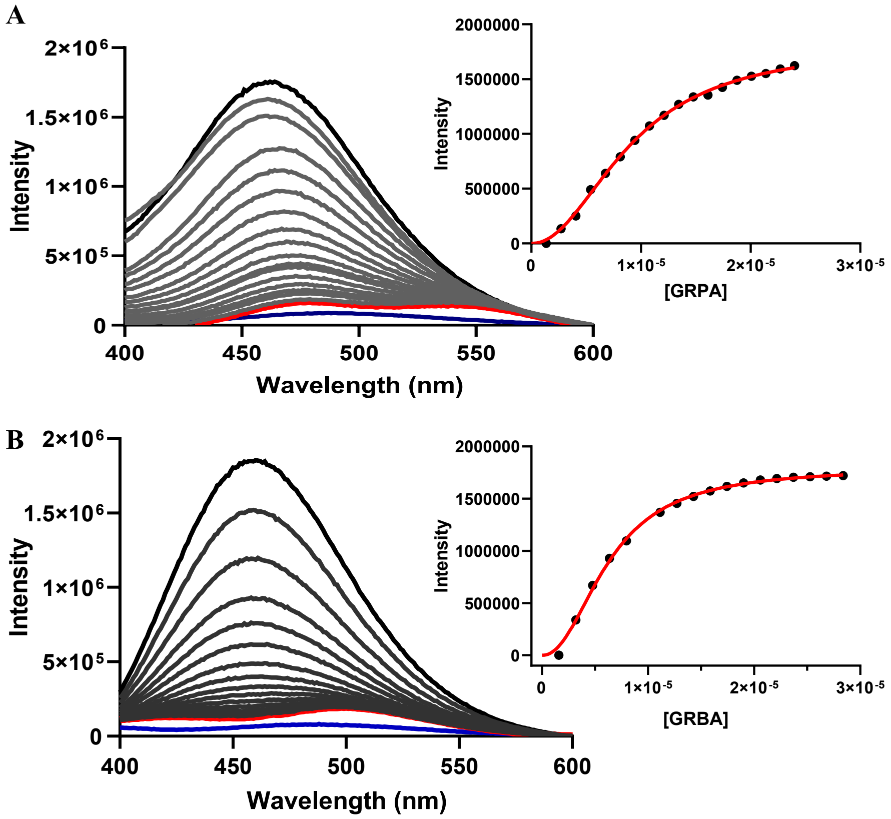

Figure S22. Emission spectrum of Hoechst (blue line) in the presence of CT-DNA (black line) at different complex concentrations (0-28 μmol L^{-1}), **GRPA** (A) and **GRB**A (B). [Hoechst] = 5 μmol L^{-1} and [CT DNA] = 10 μmol L^{-1}, λ_{exc}= 340 nm.

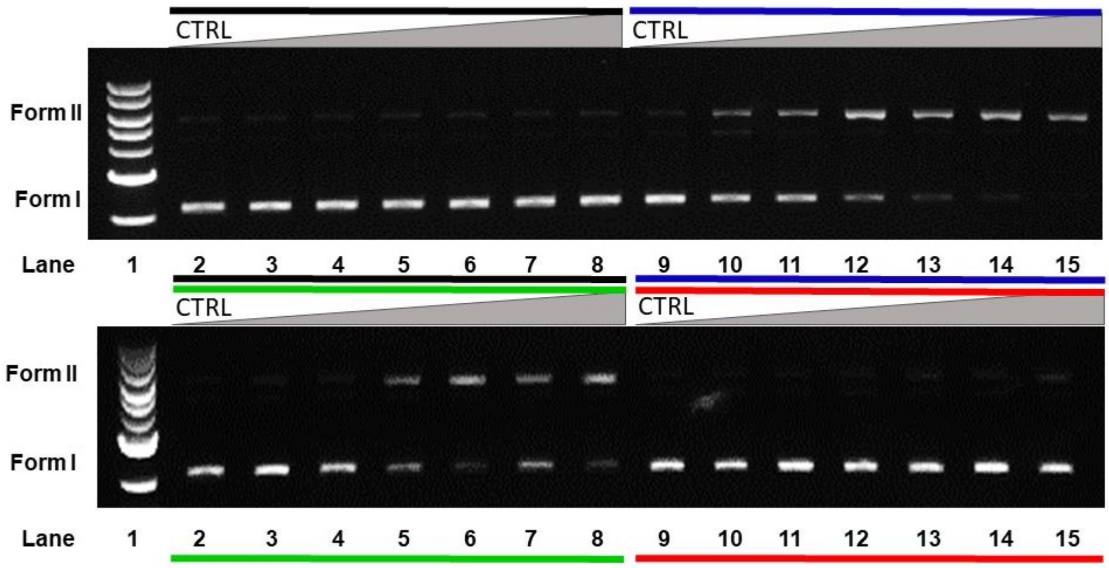

Figure S23. Photocleavage of 20 μmol L^{-1} (in base pair) pBR322 DNA in the presence of **GRBA** in the dark and after 1 h of irradiation with blue, green and red LEDs. In all experiments, lane 1 contains only linear DNA ladder and lane 2 has only pBR322 DNA, while lanes 3−8 and 10−15 contained the following concentrations of 0.5, 1.0, 3.0, 5.0, 7.0 and 10 μmol L^{-1} of **GRBA**. Dark, blue, green and red lines indicate either the experiment was carried out in the dark or with blue, green or red-light irradiation.

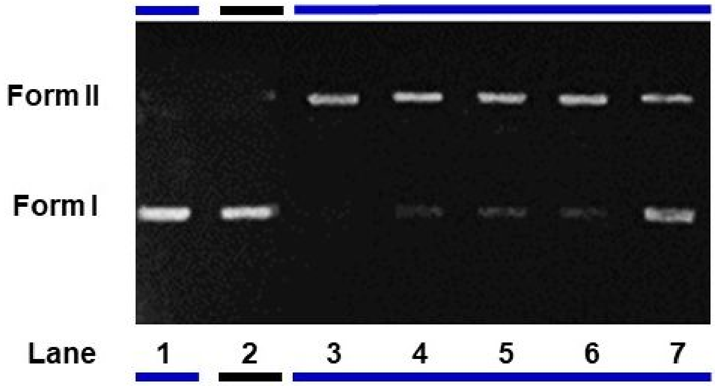

Figure S24. Photocleavage of pBR322 DNA (20 μmol L^{-1}) in the presence of **GRBA** (5 μmol L^{-1}) after 1 h of blue LED irradiation in the presence of radical scavengers. Lane 1: pBR322 DNA only with blue irradiation. Lane 2: DNA + complexes in the dark. Lane 3: DNA + complexes with blue light irradiation. Lanes 4 – 7: pBR322 DNA + **GRBA** + suppressors pyruvate (4), histidine (5), D-mannitol (6) and tiron (7), respectively.

Table S1. TD-DFT Wavelengths (oscillator strengths) and characters of the spin-allowed electronic transitions assigned of selected transitions for **GRPA** and **GRBA** in methanol media.

**λ/nm (*f*)**	**Transition (%)**	**Character**	**Assignment**
	**GRPA**		
525 (0.12)	HOMO→LUMO (98%)	anth→bpy*	ILCT
434 (0.24)	HOMO-3→LUMO (84%)	Ru→bpy*	MLCT
412 (0.083)	HOMO-3→LUMO+2 (38%)<br>HOMO-2→LUMO+1 (30%)	Ru→phen*	MLCT
373 (0.094)	HOMO-3→LUMO+4 (48%)	Ru→phen*	MLCT
272 (0.87)	HOMO-4→LUMO+6 (13%)<br>HOMO→LUMO+10 (33%)	anth→anth*	IL
257 (0.50)	HOMO-9→LUMO+2 (15%)<br>HOMO→LUMO+11 (19%)	phen→phen*<br>anth→anth*	IL
256 (0.39)	HOMO→LUMO+11(27%)<br>HOMO-12→LUMO (13%)	anth→anth*<br>bpy→bpy*	IL
	**GRBA**		
524 (0.02)	HOMO→LUMO (0.02)	anth→bpy*	ILCT
436 (0.20)	HOMO-2→LUMO (51%)<br>HOMO-3→LUMO (31%)	Ru→bpy*	MLCT
413 (0.13)	HOMO-3→LUMO+1 (43%)<br>HOMO-2→LUMO+2 (34%)	Ru→phen*	MLCT
291 (0.23)	HOMO-8→LUMO (46%)	bpy→bpy*	IL
276 (0.70)	HOMO-6→LUMO+2 (32%)<br>HOMO-7→LUMO+1 (23%)	bpy→bpy*	IL
271 (0.69)	HOMO-4→LUMO+5 (24%)<br>HOMO→LUMO+10 (30%)	anth→anth*	IL
255 (0.13)	HOMO-9→LUMO+3 (29%)<br>HOMO-8→LUMO+3 (21%)	bpy→bpy*	IL

Table S2 – Effect of **GRPA** associated with ampicillin (AMP) and tetracycline (TETRA) antibiotics against *S. aureus* and *S. epidermidis*.

**BACTERIAL STRAIN**	**RUTHENIUM COMPLEX**				**ANTIBIOTIC**				**COMBINED EFFECT**
	**GRPA**				**AMP**				
	MIC# µg/mL (µmol L^{-1}) (individual)	MIC$ µg/mL (combined)	FIC_A		MIC µg/mL (µmol L^{-1}) (individual)	MIC µg/mL (combined)	FIC_B	FICI	
S. aureus ATCC 25923	3.9 (3.41)	0.24	0.0615		0.39 (1.11)	0.024	0.0615	0.123	Synergism
S. aureus ATCC 700698	1.9 (1.66)	0.12	0.0631		50 (143.10)	25	0.5	0.563	Indifferent
S. epidermidis ATCC 12228	1.9 (1.66)	0.12	0.0631		1.56 (4.46)	0.097	0.0622	0.125	Synergism
S. epidermidis ATCC 35984	3.9 (3.41)	0.24	0.0615		1,000 (2,862)	62.5	0.0625	0.124	Synergism
	**GRPA**				**TETRA**				
	MIC µg/mL (µmol L^{-1}) (individual)	MIC µg/mL (combined)	FIC_A		MIC µg/mL (µmol L^{-1}) (individual)	MIC µg/mL (combined)	FIC_B	FICI	
S. aureus ATCC 25923	3.9 (3.41)	0.24	0.0615		0.39 (0.87)	0.024	0.0615	0.123	Synergism
S. aureus ATCC 700698	1.9 (1.66)	0.12	0.0631		100 (225)	50	0.5	0.563	Indifferent
S. epidermidis ATCC 12228	1.9 (1.66)	0.12	0.0631		0.39 (0.87)	0.097	0.248	0.311	Synergism
S. epidermidis ATCC 35984	3.9 (3.41)	0.48	0.123		0.19 (1.75)	0.024	0.126	0.25	Synergism

Individual Minimum Inhibitory Concentration (MIC), $ Combined Minimum Inhibitory Concentration (MIC), Fractional Inhibitory Concentration (FIC) and Fractional Inhibitory Combination Index (FICI) values. FICI ≤ 0.5 means synergistic effect, FICI > 0.5 ≤ 4.0 means indifferent effect, FICI > 4.0 means antagonistic effect.

Table S3 - Effect of the **GRBA** complex associated with AMP and TETRA antibiotics on *S. aureus* and *S. epidermidis*.

BACTERIAL STRAIN	RUTHENIUM COMPLEX				ANTIBIOTIC				COMBINED EFFECT
	GRBA				AMP				
	MIC[#] µg/mL (µmol L^{-1}) (individual)	MIC[$] µg/mL (combined)	FIC_A		MIC µg/mL (µmol L^{-1}) (individual)	MIC µg/mL (combined)	FIC_B	FICI	
S. aureus ATCC 25923	3.9 (3.57)	0.975	0.25		0.39 (1.11)	0.097	0.25	0.5	Synergism
S. aureus ATCC 700698	1.9 (1.74)	0.95	0.5		50 (143.10)	25	0.5	1.0	Indifferent
S. epidermidis ATCC 12228	1.9 (1.74)	0.12	0.0631		1.56 (4.46)	0.097	0.0621	0.125	Synergism
S. epidermidis ATCC 35984	3.9 (3.57)	0.24	0.0615		1,000 (2,862)	62.5	0.0625	0.124	Synergism
	GRBA				TETRA				
	MIC µg/mL (µmol L^{-1}) (individual)	MIC µg/mL (combined)	FIC_A		MIC µg/mL (µmol L^{-1}) (individual)	MIC µg/mL (combined)	FIC_B	FICI	
S. aureus ATCC 25923	3.9 (3.57)	0.24	0.0615		0.39 (0.87)	0.195	0.5	0.561	Indifferent
S. aureus ATCC 700698	1.9 (1.74)	0.12	0.0631		100 (225)	50	0.5	0.563	Indifferent
S. epidermidis ATCC 12228	1.9 (1.74)	0.12	0.0631		0.39 (0.87)	0.097	0.25	0.313	Synergism
S. epidermidis ATCC 35984	3.9 (3.57)	0.24	0.0615		0.19 (0.43)	0.097	0.5	0.561	Indifferent

Individual Minimum Inhibitory Concentration (MIC), $ Combined Minimum Inhibitory Concentration (MIC), Fractional Inhibitory Concentration (FIC) and Fractional Inhibitory Combination Index (FICI) values. FICI ≤ 0.5 means synergistic effect, FICI > 0.5 ≤ 4.0 means indifferent effect, FICI > 4.0 means antagonistic effect.

Table S4. Selectivity index (SI) of the metal compounds for different tumor cell lines through the ratio SI = IC_{50}(MRC-5)/IC_{50}(tumor cell).

SI	MDA-MB-231	A2780	A549
**GRPA**	7.1	9.3	2.3
**GRBA**	> 11.2	> 13.5	> 2.9
